# Importance of factors determining the effective lifetime of a mass, long-lasting, insecticidal net distribution: a sensitivity analysis

**DOI:** 10.1186/1475-2875-11-20

**Published:** 2012-01-13

**Authors:** Olivier JT Briët, Diggory Hardy, Thomas A Smith

**Affiliations:** 1 Department of Epidemiology and Public Health, Swiss Tropical and Public Health Institute, Basel, Switzerland; 2 University of Basel, Basel, Switzerland

**Keywords:** LLIN, Simulation, Life, Mass, Distribution, Lasting, Insecticidal, Net

## Abstract

**Background:**

Long-lasting insecticidal nets (LLINs) reduce malaria transmission by protecting individuals from infectious bites, and by reducing mosquito survival. In recent years, millions of LLINs have been distributed across sub-Saharan Africa (SSA). Over time, LLINs decay physically and chemically and are destroyed, making repeated interventions necessary to prevent a resurgence of malaria. Because its effects on transmission are important (more so than the effects of individual protection), estimates of the lifetime of mass distribution rounds should be based on the effective length of epidemiological protection.

**Methods:**

Simulation models, parameterised using available field data, were used to analyse how the distribution's effective lifetime depends on the transmission setting and on LLIN characteristics. Factors considered were the pre-intervention transmission level, initial coverage, net attrition, and both physical and chemical decay. An ensemble of 14 stochastic individual-based model variants for malaria in humans was used, combined with a deterministic model for malaria in mosquitoes.

**Results:**

The effective lifetime was most sensitive to the pre-intervention transmission level, with a lifetime of almost 10 years at an entomological inoculation rate of two infectious bites per adult per annum (ibpapa), but of little more than 2 years at 256 ibpapa. The LLIN attrition rate and the insecticide decay rate were the next most important parameters. The lifetime was surprisingly insensitive to physical decay parameters, but this could change as physical integrity gains importance with the emergence and spread of pyrethroid resistance.

**Conclusions:**

The strong dependency of the effective lifetime on the pre-intervention transmission level indicated that the required distribution frequency may vary more with the local entomological situation than with LLIN quality or the characteristics of the distribution system. This highlights the need for malaria monitoring both before and during intervention programmes, particularly since there are likely to be strong variations between years and over short distances. The majority of SSA's population falls into exposure categories where the lifetime is relatively long, but because exposure estimates are highly uncertain, it is necessary to consider subsequent interventions before the end of the expected effective lifetime based on an imprecise transmission measure.

## Background

Over the period 2008-2010, an estimated 290 million long-lasting insecticidal nets (LLINs) were distributed in sub-Saharan Africa [1]. LLINs reduce malaria transmission by protecting individuals from infectious bites, and by reducing the probability that a mosquito survives the extrinsic incubation period. Whereas continuous distribution through antenatal clinics is common, most LLINs are being distributed through mass campaigns, reaching a large proportion of the population at risk of malaria. Over time, after a mass distribution, the proportion of the population sleeping under an LLIN decreases. This is partly due to attrition (the loss of nets available for their intended use, *e.g*. by alternative use), but also due to new births and user fatigue adding to the unprotected population.

The effective protection of LLINs against mosquito bites also wanes as they decay physically (hole formation) and chemically (insecticide loss).

Many LLIN programmes work with the assumptions that there is little variability in the decay among nets and that they last about 3 years, at which time they need replacement. However, variability in net decay appears to be substantial and the average 'lifespan' could be considerably less than 3 years [2]. The number of LLINs remaining in households does not take the physical and chemical state of the nets into account, and a proportion of those nets may have lost considerable functionality [3]. The World Health Organization (WHO) recommends tracking the physical integrity of nets (number, size and location of holes) and the insecticidal activity, measured by knock down and killing in standard WHO cone and tunnel tests [2]. Unfortunately, little is known about how these quantities, alone or in interaction, affect personal protection, and how they could be used to define when a net is worn out and at the end of its 'useful life'.

Even if individual nets do not adequately prevent mosquitoes from inoculating the user, they may still reduce mosquito survival and thus affect transmission at the population level. This community effect is likely to be more important than personal protection in preventing inoculations [4,5]. The timing of repeat LLIN distributions may also depend on the characteristics of the human population, in particular the transmission level and immune status. Whereas knowing the 'useful life' of individual LLINs (for which a cut-off minimum functionality would need to be defined, below which an LLIN would be declared 'dead') might facilitate planning in continuous distribution programmes. For a round of mass distributed LLINs, the 'effective lifetime', based on the duration of the malaria preventive effect at population level capturing all the effects described above, might be more useful for planning the timing of subsequent rounds.

This paper describes a simulation experiment to predict the duration of epidemiological protection offered by a mass LLIN distribution targeting the general population and identifies the factors that are important in determining it.

## Methods

The OpenMalaria modelling platform [6] is an open source C++ programme and takes scenario specification inputs in eXtensible Markup Language (XML). In this platform, stochastic individual-based models for malaria in humans are combined with a deterministic model for malaria in mosquitoes, which have been fitted to multiple field data sets [7]. For each five-day time step, data on a human population is updated via components representing new infections, parasite densities, acquired immunity, uncomplicated and severe malaria episodes, direct and indirect mortality, infectiousness to mosquitoes, and case management. Each simulated malaria infection has a distinct parasite density that varies by time step, while the malaria transmission level varies seasonally. The models can accommodate multiple mosquito species with varying periodical emergence rates, and non-human hosts [8,9]. An ensemble of 14 model variants [10] is currently available, capturing a range of possibilities for the dynamics of malaria in humans.

For this experiment, the existing insecticide treated net (ITN) intervention model component [8,9] was developed to include capability to model physical and chemical decay of LLINs. The effect of LLINs, depending on their physical and chemical state, on deterrence, pre-prandial and post-prandial killing of malaria vector mosquitoes (see Appendix) was parameterised, using published experimental hut data [11-14].

**Figure 1:**
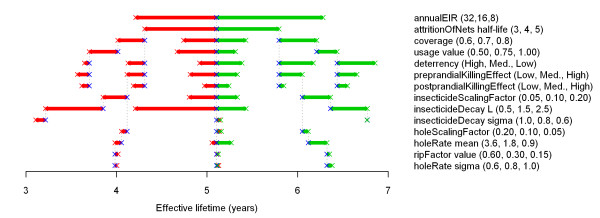
**'Skeleton' diagram of model parameters**. For each parameter or parameter group listed, the outcome (effective lifetime of a mass LLIN distribution, in years) depending on the parameter value is plotted on the horizontal axis. The effective lifetime was defined as the length of the period since mass distribution that the number of prevented episodes was above half the value for the year with maximum impact (*i.e*. the year with the minimum number of episodes), as compared to a scenario without any intervention. For each parameter, three values were chosen (listed in parenthesis after the parameter name), which represent roughly the lower, central, and upper values of the plausible parameter range. The central value is not necessarily the mean of the extreme values. These parameter values are listed in order of effect on the outcome; the first parameter value has the lowest associated outcome value, the last parameter has the highest. Crosses represent models; identical models are connected with dotted black vertical lines. Blue crosses indicate models with the central parameter value listed, green crosses indicate models with an extreme parameter value with an associated high outcome, and red crosses indicate models with an extreme parameter value with an associated low outcome. Red lines connect red and blue crosses, green lines connect blue and green crosses. In addition to results from models that vary only the parameter value in question (the other parameters taking the central value), outcomes for selected parameter combinations, often contingent on each other, where the selected parameters all have the values with the lower (red crosses), or higher (green crosses) associated outcomes are plotted.

Outcomes based on the incidence of all-age uncomplicated and clinical malaria episodes were considered, which is the measure of the malaria burden most easily accessible in control programmes. Full economic analysis should weight severe and fatal episodes more heavily and take age into account, but in these models there is considerable uncertainty about predicted morbidity rates in older age groups. The following measure of effective lifetime was used: the length of the period since mass distribution during which the number of prevented episodes was above half the numerical value of the year with maximum impact on malaria episodes (*i.e*. the year with the minimum number of malaria episodes), as compared to a scenario without any intervention. A sensitivity analysis was done on how the effective lifetime of a mass LLIN distribution depended on the pre-intervention entomological inoculation rate (EIR) (which was varied by scaling vector emergence), initial coverage, attrition rate, LLIN effects, and physical and chemical decay rates and other LLIN-related parameters. Each of 14 parameters or parameter groups (listed in Figure 1 and discussed in the Appendix) were varied over three values (low, central and high), while keeping all other parameters and parameter groups at their central values (see Additional file 1 and the link [15] for an intervention scenario with all parameters at central value).

Also, selected parameter and parameter group combinations that seemed likely to be interdependent or to have multiplicative effects (Figure 1), were varied. For each combination, parameter values were chosen that acted on the outcome in the same direction. This was done both with those parameter values in the combination associated with lower outcomes, and with those values associated with higher outcomes. The parameters outside the combinations were kept at central values.

The relationship between the effective lifetime and pre-intervention EIR was studied in more detail by varying it over a wider range, together with LLIN attrition half-life. The effect of initial coverage was also looked at in more detail. Because the cut-off at half the impact on malaria episodes in the definition of the effective lifetime is arbitrary, sensitivity to this was studied. Finally, whether or not the protective epidemiological impact could be sustained in hypothetical situations where coverage is sustained throughout and where nets do not decay, depending on the pre-intervention EIR and coverage, was explored.

## Results

**Figure 2:**
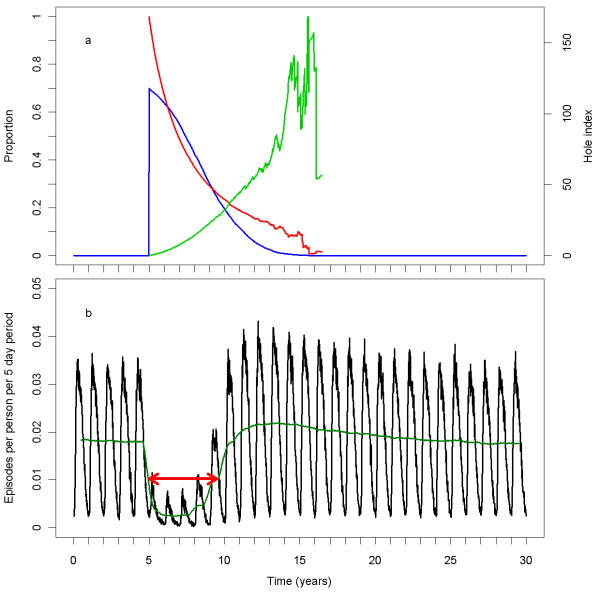
**Central scenario simulation with base model**. a) The blue line (on left vertical axis) represents the proportion of the population covered, the red line (on left vertical axis) represents the mean insecticide in the remaining LLINs as a proportion of its initial value. The light green line (on the right vertical axis) represents the mean hole index in the remaining LLINs. b) The black line represents the number of episodes per person per five-day period. The dark green line represents the 1 year moving average of the number of episodes per person per five-day period. The red arrow indicates the approximate length of the effective lifetime of the LLIN distribution.

Figure 2 illustrates a simulation run of the central scenario with the base model (*R0000*). At the beginning of the sixth year of the simulation, LLINs were assigned to 70% of the population. Over time, the coverage declined, the insecticide in the remaining LLINs declined, and the hole index in the remaining LLINs increased (Figure 2a). Because of variation in the rates of insecticide loss and hole formation, the curves for the mean hole index and mean insecticide content became erratic as fewer LLINs remained. In the absence of intervention, the number of episodes per person per five-day time step (Figure 2b) reflects the annual periodicity of the vector emergence. The effect of an LLIN distribution is best illustrated by the de-seasonalised trend. Immediately after LLIN assignment, the number of episodes declined, reaching a minimum after two years. The number then rose with an S-shaped curve, reaching a slightly higher level than pre-intervention, and declined gradually to the pre-intervention level. The red arrow in Figure 2b illustrates the approximate point where the impact of the LLIN distribution round is half of its maximum, and the time from distribution until that point.

The results of the sensitivity analysis are plotted in a 'skeleton' diagram (Figure 1), which is an adaptation and expansion of a tornado diagram. A skeleton diagram not only displays how strongly the outcome varies with each parameter over its tested range, with all other parameters at their central values; it also indicates, for selected parameter combinations only, how the sensitivity to a parameter is altered if other parameters on which the parameter in question might be contingent are at their extremes.

Effective lifetime appears to be particularly sensitive to three parameters: pre-intervention EIR (*annualEIR*), attrition of nets (*attritionOfNets half-life*) and half-life of insecticide decay (*insecticideDecay L*).

The skeleton diagram shows that, for example, the bar lengths for *coverage*, *deterrency*, *preprandialKillingEffect*, and *postprandrialKillingEffect *are shorter for the centre-to-low (red) bars connected to the lower extreme for *attritionOfNets half-life *(red cross), than for those connected to the central level of *attritionOfNets half-life *(blue cross).

Because the attrition rate determines in part how many nets are left (together with the initial receipt proportion, defined by the parameter *coverage*), it strongly interacts with parameters that describe the LLIN effects on the vector population. At the lower extremes of both *attritionOfNets half-life *and *preprandialKillingEffect*, the centre-to-low (red) bar for the *insecticideScalingFactor *is only slightly shorter than the centre-to-low (red) bar with both *attritionOfNets half-life *and *preprandialKillingEffect *(and all other parameters) at central level. Further, one level deeper, at the lower extreme of the *insecticideScalingFactor*, the length of the centre-to-low (red) bar for *insecticideDecay L *is shorter than that with all other parameters at central level, yet still important. From Figure 1, it is not possible to establish how much the variation in each of the parameters for *attritionOfNets half-life*, *preprandialKillingEffect*, and *insecticideScalingFactor *contributes to the shortening of the *insecticideDecay L *bar. However, it is likely that *attritionOfNets half-life *has an important role, as insecticide decay only acts on remaining nets and is thus strongly contingent on the attrition rate. At a long attrition half-life, the sensitivity to the mean insecticide decay rate and its 'downstream' parameter (the parameter for variation in insecticide decay is contingent on the mean insecticide decay rate) will be much stronger than at a shorter attrition half-life because most nets will disappear in the latter scenario before the decay rate matters much. This is similar for the mean hole formation rate and its 'downstream' parameters (the parameters for variation in the hole formation rate and the rip factor are contingent on the mean hole formation rate). The *holeRate mean *is more important at the higher extreme of *attritionOfNets half-life*, *preprandialKillingEffect*, and *holeScalingFactor *than with all parameters at central level. Nevertheless, the outcome appears to be insensitive to parameters determining physical decay (hole formation).

**Figure 3:**
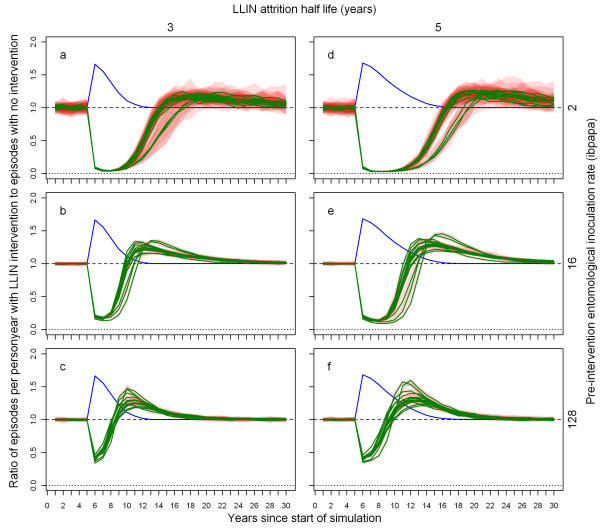
**Ratio of episodes per year with LLIN mass distribution to episodes with no intervention, depending on pre-intervention EIR, attrition half-life and model variant**. Each green line represents the median number of episodes of 10 simulation runs (each with unique random seed) with LLINs distributed to 70% of the people (population size = 10,000) at the beginning of year 6, the number of episodes in each intervention simulation run divided by the number of episodes in a simulation run without LLINs (also with unique random seed). Even though the values are annual totals, they are connected with lines. A column graph would be more appropriate, but less convenient for plotting multiple data series. The red semi transparent polygons represent the range of the 10 runs. Per panel, there are 14 green lines (and 14 red polygons), each representing a malaria model variant. The first column of panels (a-c) shows results for attrition with a three year half-life and the second column (d-f) for attrition with a five year half-life. The panel rows show results for pre-intervention EIRs of two (a & d), 16 (b & e), and 128 (c & f) infectious bites per adult per annum (ibpapa). Blue lines represent the proportion of people using an LLIN, added to one. Horizontal lines: dashed, ratio = 1; dotted, ratio = 0.

**Figure 4:**
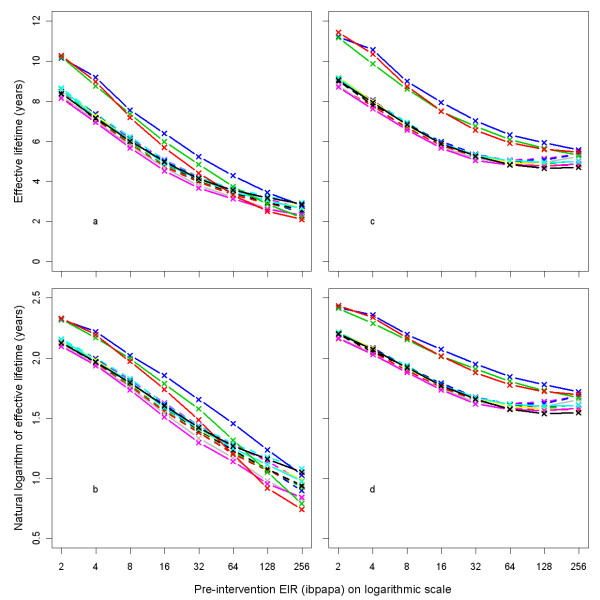
**Effective lifetime of a mass LLIN distribution, depending on model variant, and pre-intervention EIR**. Effective lifetime was defined as the length of the period since mass distribution during which impact on malaria episodes was above half the numerical value of the year with maximum impact (*i.e*. the year with the minimum number of episodes), as compared to a scenario without any intervention. Impact was measured in (a & b) all-age uncomplicated and complicated malaria episodes prevented, or (c & d) in all-age infections prevented. The entomological inoculation rate (EIR) was defined in infectious bites per adult per annum (ibpapa). Model variants [10]: R0000 = solid black lines and black crosses; R0063 = solid red lines and red crosses; R0065 = solid green lines and green crosses; R0068 = solid blue lines and blue crosses; R0111 = solid light blue lines and light blue crosses; R0115 = solid magenta lines and magenta crosses; R0121 = solid yellow lines and yellow crosses; R0125 = solid grey lines and grey crosses; R0131 = dashed black lines and black crosses; R0132 = dashed red lines and red crosses; R0133 = dashed green lines and green crosses; R0670 = dashed blue lines and blue crosses; R0674 = dashed light blue lines and light blue crosses; R0678 = dashed magenta lines and magenta crosses.

Figure 3 illustrates how, depending on the pre-intervention EIR and attrition half-life, the ratio of malaria episodes per year for a situation with LLINs to a situation without LLINs develops over time. In general, the number of episodes reaches a minimum a few years after distribution that is slightly lower than in the first year after distribution, and then increases with an S-shape, becoming larger than one (indicating more episodes in the intervention situation) before gradually dropping to one. With increasing pre-intervention EIR, the minima are not as low, the subsequent rise in episodes less steep, and the maxima are higher. With a lower attrition rate (longer half-life of 5 years) the graphs appear to be horizontally stretched versions of the situation with higher attrition (shorter half-life of 3 years). At a low pre-intervention EIR, the impact on malaria episodes lasts until most LLINs have disappeared. At a high pre-intervention EIR, there are still a large number of nets (albeit with holes and less insecticide) present when the number of episodes surpasses the pre-intervention level. At higher pre-intervention EIRs, the simulation run ranges are very narrow, indicating that there is little stochasticity (and that a population size of 10,000 is appropriate). Particularly for the lower pre-intervention EIRs, three model variants, *R0063*, *R0065 *and *R0068*, which vary heterogeneity in human exposure [10], yield results that are different from the 11 other variants, with longer lasting LLIN effects. This is illustrated by Figure 4, which shows how the effective lifetime outcome depends on pre-intervention EIR and model variant, at an attrition half-life of four years. If effective lifetime is defined based on the incidence of infections (Figure 4c &4d) instead of episodes (Figure 4a &4b), then lifetime is consistently higher for these model variants than for the base model. For episodes, however, the effective lifetime with model *R0068 *converged with that of the base model *R0000 *at high pre-intervention EIR levels, and the lifetime of models *R0063 *and *R0065 *decreased more sharply with increasing pre-intervention EIR than with the base model *R0000*.

**Figure 5:**
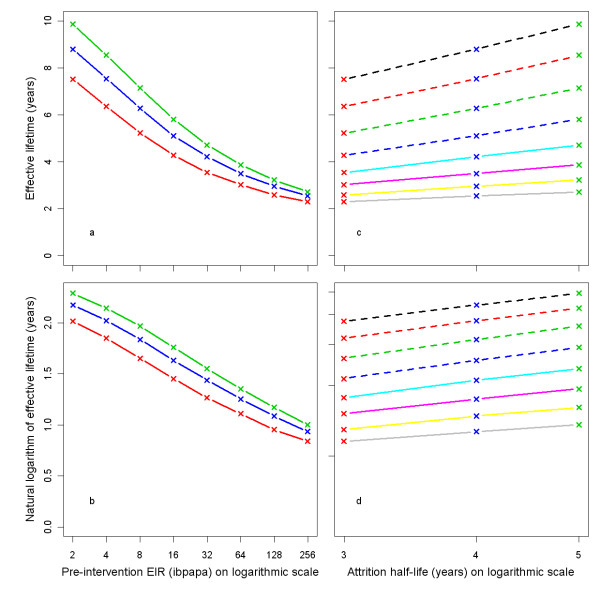
**Effective lifetime of a mass LLIN distribution, depending on pre-intervention EIR and attrition half-life**. Entomological inoculation rate (EIR) is defined as infectious bites per adult per annum (ibpapa). Attrition half-life (years): red lines and crosses = 3, blue lines and crosses = 4, green lines and crosses = 5. Figures c & d EIR: dashed black lines = 2, dashed red lines = 4, dashed green lines = 8, dashed blue lines = 16, light blue lines = 32, magenta lines = 64, yellow lines = 128, grey lines = 256. Figures a & c) Effective lifetime on vertical axis, Figures b & d) Natural logarithm of effective lifetime on vertical axis.

Effective lifetime, averaged over all 14 model variants, is plotted against the pre-intervention EIR in Figure 5a. The relationship follows approximately a straight line if the outcome is logarithmically transformed (Figure 5b). The effective lifetime is linearly related to the logarithm of the attrition half-life (Figures 5c and 5d).

**Figure 6:**
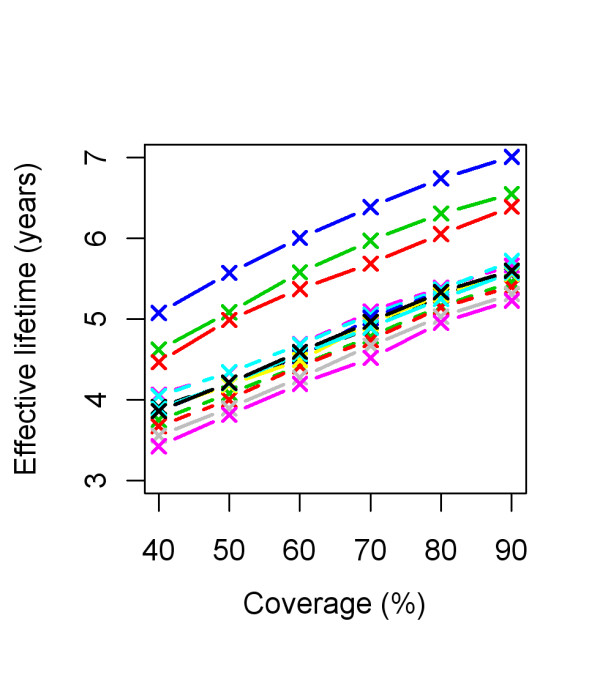
**Effective lifetime of a mass LLIN distribution, depending on initial coverage and model variant**. Initial coverage is the percentage of people that received access to an LLIN at the time of distribution. All parameters other than initial coverage are at central values. Model variants are coloured as indicated in Figure 4.

Figure 6 illustrates how effective lifetime depends on initial coverage. This appears to be a linear relationship with the proportion of the population having access to an LLIN. As the lines for model variants are largely parallel to one another, there is little interaction between model specification and initial coverage over the range studied.

**Figure 7:**
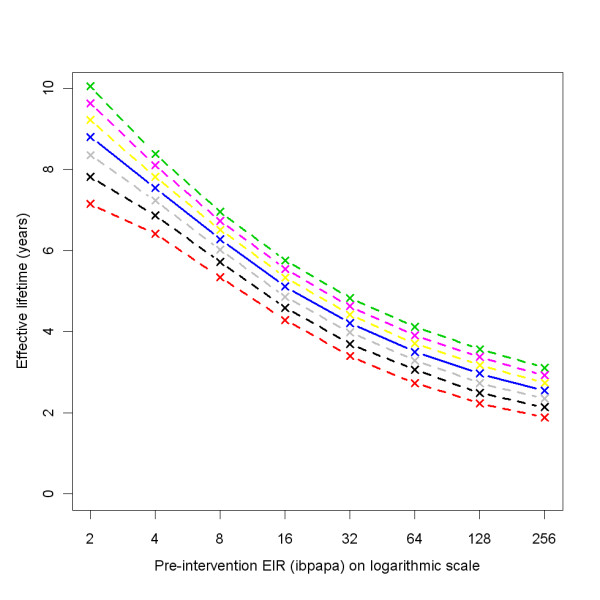
**Effective lifetime of a mass LLIN distribution, depending on lifetime definition, and pre-intervention EIR**. Entomological inoculation rate (EIR) is defined by infectious bites per adult per annum (ibpapa). Effective lifetime is defined as the length of the period since mass distribution during which the number of prevented all-age uncomplicated and complicated malaria episodes was above a set proportion of the numerical value of the year with maximum impact (minimum number of episodes), as compared to a scenario without any intervention, with proportions set at 0.2 (dashed green line), 0.3 (dashed magenta line), 0.4 (dashed yellow line), 0.5 (solid blue line), 0.6 (dashed grey line), 0.7 (dashed black line), or 0.8 (dashed red line).

The sensitivity of the result to the definition of effective lifetime is illustrated in Figure 7. The blue line is identical to the blue line in Figure 5a, representing the relationship between effective lifetime and pre-intervention EIR at a central value attrition half-life of 4 years. The result appears insensitive (relative to the effect of pre-intervention EIR) to the cut-off impact level in effective lifetime.

**Figure 8:**
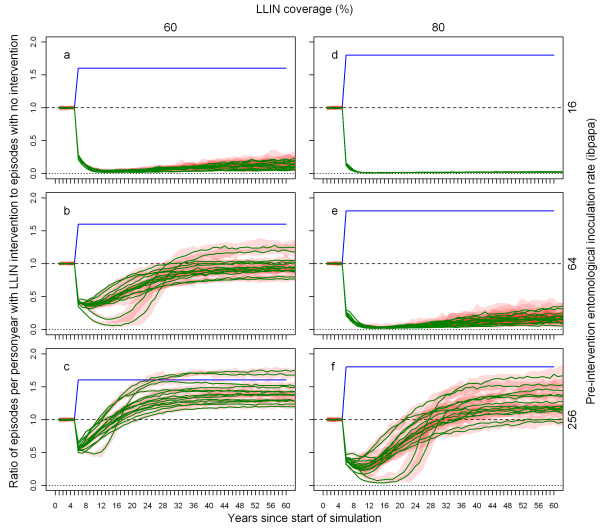
**Ratio of annual episodes in a situation with LLIN sustained coverage without attrition or decay to a situation without LLINs, depending on pre-intervention EIR and coverage**. Each green line represents the median number of episodes of 10 simulation runs (each with unique random seed) with LLINs distributed to the people (population size = 10,000) at the beginning of year 6, the number of episodes in each intervention simulation run divided by the number of episodes in a simulation run without LLINs (also with unique random seed). The red semi transparent polygons represent the range of the 10 runs. Per panel, there are 14 green lines (and 14 red polygons), each representing a malaria model variant. The first column of panels (a-c) shows results for 60% coverage and the second column (d-f) for 80% coverage. The panel rows show results for pre-intervention EIRs of 16 (a & d), 32 (b & e), and 256 (c & f) ibpapa. Blue lines represent the proportion of people using an LLIN, added to one. Horizontal lines: dashed, ratio = 1; dotted, ratio = 0.

Results from the computing experiment where LLIN coverage was sustained and where nets did not decay over time are presented in Figure 8. With 60% coverage, at low pre-intervention EIR of four infectious bites per adult per annum (ibpapa) LLINs suppressed transmission almost fully, with clinical episodes occurring almost exclusively as a result of imported infections (see Appendix). At the intermediate pre-intervention EIR of 16 ibpapa, the number of episodes slowly increased following a maximum impact about 10 years after the initial distribution. At the higher pre-intervention EIR of 64 ibpapa, the number of episodes increased at a much higher rate, and 25 years after the start of the intervention, it appeared to approach a new equilibrium below the pre-intervention level. The point at which half of the maximum impact level was crossed took place 17.9 years after distribution. At the highest pre-intervention EIR of 256 ibpapa, the increase was even faster reaching a new equilibrium with effectively more clinical episodes than in a situation without LLINs, and the point at which half of the maximum impact level was crossed occurred 5.6 years after distribution. With 80% coverage, the results were somewhat similar, yet shifted by one EIR category (EIR multiplied by four). The plot for 80% coverage at pre-intervention EIR = 256 ibpapa is somewhat similar to that for 60% coverage at pre-intervention EIR = 64 ibpapa. With 80% coverage, only at pre-intervention EIR = 256 ibpapa was the half of maximum impact level crossed, 15.6 years after distribution.

**Figure 9:**
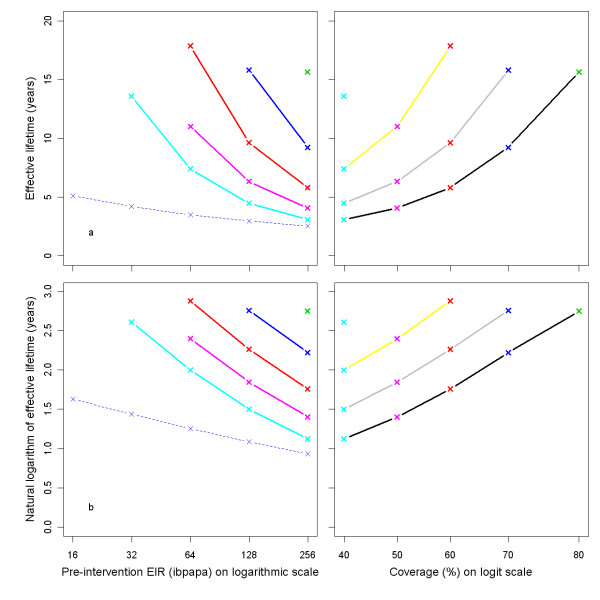
**Effective lifetime of LLIN sustained coverage, depending on pre-intervention EIR and coverage**. Entomological inoculation rate (EIR) is defined by infectious bites per adult per annum (ibpapa). Coverage (%): light-blue lines and crosses = 40, magenta lines and crosses = 50, red lines and crosses = 60, blue lines and crosses = 70, green crosses = 80. Figures c & d EIR: yellow lines = 64, grey lines = 128, black lines = 256. Figures a & c) Effective lifetime on vertical axis, Figures b & d) Natural logarithm of effective lifetime on vertical axis. For comparison, the effective life of a mass distribution with 70% coverage and a half life of four years is plotted in a and b (thin dashed blue lines and crosses).

Results in terms of the effective lifetime for all pre-intervention EIRs and coverage level combinations tested are displayed in Figure 9. For some combinations, in one or more simulations, the impact did not fall to less than half of the maximum, thus effective lifetime could not be calculated. The effective lifetime for a single mass distribution at 70% coverage (thin dashed blue line) can be directly compared to the lifetime of sustained 70% coverage (blue line).

**Figure 10:**
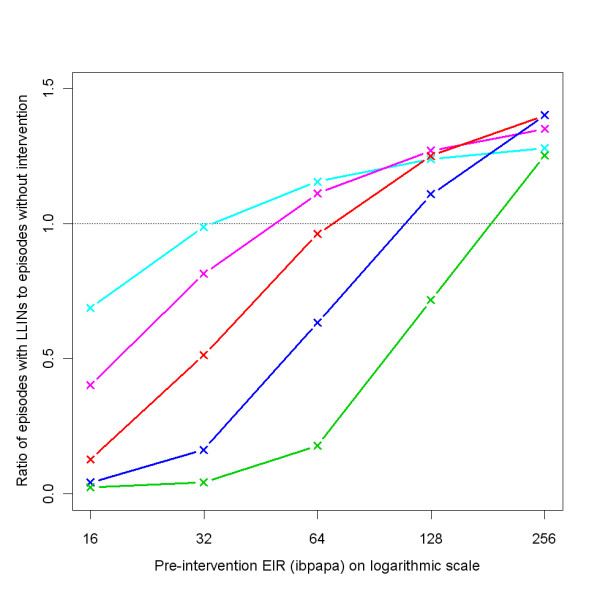
**Equilibrium ratio of episodes with LLIN sustained coverage to episodes without intervention, depending on pre-intervention EIR and coverage**. Coverage (%): light blue (40), magenta (50), red (60), blue (70), green (80). The dotted horizontal line indicates a ratio of 1.

Figure 10 shows the equilibrium ratio of episodes in situations with sustained LLIN coverage to episodes without intervention, depending on pre-intervention EIR and coverage. At 80% coverage, only at the highest EIR is the post-intervention equilibrium above one.

## Discussion

### Effective lifetime

To plan the timing of malaria control interventions after a mass LLIN distribution, it would be useful to understand how long the protective effect of the LLIN distribution will last. Following intervention, the impact on clinical malaria incidence reaches a maximum within a short period and is sustained for some time, after which the number of episodes rises steeply. Clearly, re-intervention (LLIN distributions or other) should take place before case incidence exceeds pre-intervention levels (due to the loss of acquired immunity). The 'effective lifetime', defined as the period from intervention until the reduction in incidence falls to a set cut-off proportion of the maximum impact, can be used to decide when to re-intervene. Because of the sharp rise in incidence it matters little whether the cut-off is set at 40, 50 or 60% of the maximum, and there is little interaction between the cut-off value and the effect of the pre-intervention EIR, indicating that the sensitivity analysis would probably give similar results if a cut-off other than 50% had been used. Even though optimal criteria for determining when to re-intervene would include economic factors, the use of the 'effective lifetime' thus provides a reasonably robust alternative.

### Pre-intervention EIR level

LLINs have approximately^a ^the same proportionate effect on vectorial capacity at different pre-intervention EIRs. However, the apparent rate at which malaria transmission resurges from a level lower than its steady state is strongly positively related to vectorial capacity. High resurgence rates appear to be associated with shallower minima in the annual number of episodes and in the annual number of (asymptomatic) infections, but this observation results mainly from temporal smoothing.

Depending on the pre-intervention EIR, three model variants showed very different effective lifetimes from the base model *R0000*. These are models *R0063*, *R0065*, and *R0068*, in which the age-specific susceptibility is independent of exposure (contrasting with the sigmoidal relationships with exposure in the other variants), and that include extra-Poisson variation in the probability of being bitten. Model *R0063 *assigns most of this to inter-host variation; model *R0065 *is intermediate, and model *R0068 *assigns the variation predominantly to 'within host variation', assuming that some individuals are always more likely to get bitten than others [10]. An explanation for why these model variants have different results is outside the scope of this paper. The models R0063 and R0065, both with inter-host variation in exposure, showed a stronger relationship between effective lifetime and pre-intervention EIR than did the base model. Heterogeneity in exposure to infectious bites between individuals is highly likely, as there are differences in mosquito access to houses [16] (depending on house quality and geographical situation) and in individual attractiveness to mosquitoes [17,18]. The effect of pre-intervention EIR is thus possibly even stronger than shown in the sensitivity analysis, based on averages of 14 model variants, 12 of which ignore inter-host variation in exposure.

For the sake of simplicity, results in the sensitivity analysis were averaged over 14 model variants. This can be problematic if there is strong interaction between the effect and the model over the range studied, as was the case with the pre-intervention EIR. Sensitivity to coverage, however, was similar for all model variants. Although the 14 model variants reflect a plausible range of models, they are not necessarily evenly spread out over this range. In order to calculate a mean effect, models should ideally be weighted based on their overall fit, and on correlations between both structure and parameter values with other variants in the ensemble. Model variants with a poor fit, and/or similar to other variants included in the analysis, should receive a low weight. To perform such a weighting is a challenging task and there is a need for more methodological development in this area to address the problem appropriately.

In all the model variants, pre-intervention EIR was varied by scaling vector emergence rates. The pre-intervention EIR could possibly also be varied by changing other variables influencing vectorial capacity, such as pre-intervention survival rate, and such a pre-intervention EIR may have a somewhat different relationship with the LLIN effective lifetime.

Vector emergence was modelled as a fixed repeating seasonal pattern, independent of the adult vector population size. If the models were to include feedback from the reduced vector population due to interventions, leading to fewer emerging mosquitoes, then longer effective lifetimes would be expected, especially at locations with lower emergence rates (where local extinction of vectors might occur). Therefore, these models give a conservative estimate of effective lifetime and its dependency on emergence rates.

### LLIN decay

Effective lifetime is highly sensitive to the attrition rate. With a short attrition half-life, nets disappear before net decay can have much impact. Hole formation and insecticidal content decay are therefore of more importance with slower attrition rates. In turn, variability in hole rate and insecticide decay rate, hole size (rip factor) and the effects of decayed nets on mosquito biting and survival, are only important when hole formation and insecticidal content decay are themselves important. Thus, the shorter the attrition half-life, the more important is its accuracy relative to that of the other net decay parameters.

Insecticide decay rate is conditional on slow attrition and one of the most important factors determining the effective lifetime of an LLIN distribution. Lifetime varies strongly between the lower half-life estimate (0.5 years, characteristic for first generation LLINs) and the central value (1.5 years, characteristic for second generation LLINs). Insecticide half-life is strongly product-dependent and the values for specific products are relatively well-defined, so, despite its importance, insecticide decay is not a major contributor to uncertainty in effective lifetime, provided that mosquitoes remain sensitive to the active compound. It remains to be studied how sensitive the effective lifetime of LLINs will be to this parameter in the presence of pyrethroid resistance [19].

Effective lifetime of an LLIN distribution was surprisingly insensitive to parameters specifying the hole formation process in the nets. It is possible that the guessed values for the *holeScalingFactor*, not based on any data, were unreasonable, and that the effect of LLINs on mosquitoes wanes much faster than presumed as the hole index increases. Even though insensitive to hole parameters, it would still be useful to have evidence-based estimates as, with the emergence and spread of pyrethroid resistance [20-22], tear resistance is expected to gain in relative importance.

### Coverage

Coverage targets can be varied relatively easily in mass distribution campaigns, thus the effects of coverage were examined in more detail. But, effective lifetime was found to be insensitive to the initial coverage, expressed as the percentage of people that had access to an LLIN at the time of distribution. Since simulated people were not grouped into households, where they could share commonly owned nets, each covered person was simulated independently with a distinct net, used every night. This precluded explicit modelling of the distinction between household ownership of nets and personal net use, net transfer patterns within families, or local protective effects shared between net users and non-users [23]. Thus the simulated coverage is equivalent to the percentage of people that used an LLIN. However, the insensitivity of effective lifetime to coverage implies that it would also be insensitive to measures of usage or familial correlations in ownership or usage.

The simulation experiment with sustained net coverage sheds some light onto what might happen if mass LLIN distributions are repeated at regular intervals, or supplemented by LLIN distribution through continuous delivery channels, such as the Expanded Program on Immunization (EPI). Although the assumption of no net attrition or decay is unrealistic, this experiment allows to distinguish the effects of attrition and decay from the transient dynamics induced by reducing exposure.

In settings with a medium to high pre-intervention EIR, after initial reduction the episode incidence will increase over time even in the absence of decay in number and state of LLINs, and reach a new equilibrium that is higher than its minimum. This is due to a reduction in the equilibrium level of acquired immunity caused by the reduction in exposure. The rate of decline in prevented episodes with sustained coverage is relatively slower than with a mass distribution with a four-year attrition half-life and central values for decay of both net coverage and physical and chemical states of the nets. For instance, at pre-intervention EIR of 128 ibpapa, with 70% coverage, for sustained coverage and for a mass distribution, the effective lifetimes were 15.8 and 3.0 years, respectively. This suggests that a decline in natural immunity has little impact on the effective lifetime of a single mass LLIN distribution.

Over a long period, lower acquired immunity levels will reduce the number of clinical episodes prevented by LLINs and will be accompanied by a shift in the age distribution of the clinical episodes. Some of the simulations suggest that prolonged low coverage levels in areas with a high pre-intervention EIR could lead to an increase in incidence that exceeds pre-intervention levels. However, many of the episodes in these simulations occur in older children or adults, and may well be milder than had they occurred at younger ages, resulting in overall lower case fatality. Nevertheless, these results suggest that a sustained high level of coverage of 80% could continue to suppress episodes at medium pre-intervention EIRs of 128 ibpapa and below, despite (complete) loss of acquired immunity. This supports high coverage targets for long-term sustained intervention planning. However, a separate simulation study involving multiple distribution mechanisms, such as antenatal care and EPI continuous distribution and repeated mass distributions, would be required before answering the question "what target coverage is most cost effective?".

## Conclusions

The strong dependency of the effective lifetime of an LLIN mass distribution on pre-intervention transmission indicates that the required distribution frequency may vary more with the local entomological situation than with LLIN quality or the characteristics of the distribution system. This highlights the need for monitoring malaria before and during intervention programmes, particularly since there are likely to be strong variations between years and over short distances. The majority of sub-Saharan Africa's population probably falls into exposure categories where the effective lifetime is relatively long [24], but because exposure estimates are highly uncertain, it is necessary to consider subsequent control measures sooner than at the end of the expected effective lifetime based on an imprecise measure of transmission.

## Endnotes

^a^Only different death rates might influence LLIN ownership, and thus LLIN effect on the vectorial capacity.

## Competing interests

The authors declare that they have no competing interests.

## Authors' contributions

OJTB designed the experiments, analysed results and drafted the manuscript. DH programmed the simulation software. TAS conceived of the study and participated in the design. All authors read and approved the final manuscript.

## Appendix

### Experiment parameterisation of ITN effects in OpenMalaria and experiment parameter values

In this Appendix, for selected parameters (italicised) and parameter groups (italicised) important for this study, detailed information is given on the choice of the parameter values.

The discussion of the parameters and parameter groups is organised according to the hierarchical organisation of a scenario script. The experiment's 'central' scenario specification in the machine readable language XML is given as Additional file 1, and also on the OpenMalaria site [15]. This XML scenario is richly annotated to explain what function the parameters have. Additional information on the function of the parameters is documented in the wiki section of the project webpage [6]

#### demography

The 'Ifakara' *demography *[25] was used. The population is stationary and approximately stable: individuals move up in age group with time, and because this structure is monotonically decreasing with age, surplus individuals are out-migrated (also above *maximumAgeYrs*).

#### popSize

A population size (*popSize*) of 10,000 was used. This is a balance between computational effort (which increases with larger population size) and the level of stochasticity (which decreases with larger population size). At a size of 10,000, the effect of rounding integers in the population demography (which is noticeable below a size of 5,000) is minimal.

#### monitoring

For this study, the following output variables are relevant: *nets owned*: the total number of nets (irrespective of physical and chemical state) present in the population; *nUncomp*: the number of uncomplicated malaria episodes; *nSevere*: the number of complicated, severe malaria episodes; *nNewInfections*: the number of new infections.

#### interventions > ITN > usage value

Usage represents the proportion of time during the night that a net is used by a simulated individual. It is not the proportion of people that use a net conditional on ownership. Because, in the current model, mosquito species bite homogenously throughout the night, the *usage value *can also be interpreted as the probability that host searching occurs during the time that people who own a net are using the net. In literature, this is called the 'π_i _value' [26]. Govella and colleagues [27] define it as "the proportion of normal exposure of unprotected humans lacking nets that occurs at times and places when net users would be protected by sleeping under them". The current model version (schema 29) allows only one *usage value *to be set, thus it is not possible to vary the π_i _value for different species within the same scenario through the *usage value*, nor can the *usage value *be varied over net users.

In this experiment, all mosquitoes were assumed to display the same host searching behaviour, with a fixed probability to search in places during times when people are protected by a net (if they own one). This, as opposed to situations where the mosquito population might be, to a degree, divided into sub-populations which either always or never search only during times and in places when people are protected by a net. Such separate behaviour could be caused by genetics, and by learning; repeating the behaviour of whatever happened in the first feeding cycle.

Comparisons of indoor *versus *outdoor human landing catches throughout the night, combined with studies of human behaviour and the source of blood meals, give insights into the proportion of host searching mosquitoes that would encounter an ITN protected host (given ownership). However, the degree to which sub-populations exist that display different behaviours is unknown.

This parameter was varied for the sensitivity analysis. A *usage value *of 0.75 was used as the central value, assuming high endophagy (the propensity to bite indoors) and biting peaks after average bed-time of the population. The extreme low parameter value was taken as 0.5 and the extreme high value was taken as 1.0. These values are based on the π_i _values reported by Govella and colleagues [27] and Russell and colleagues [28].

#### interventions > ITN > holeRate mean

**Figure 11:**
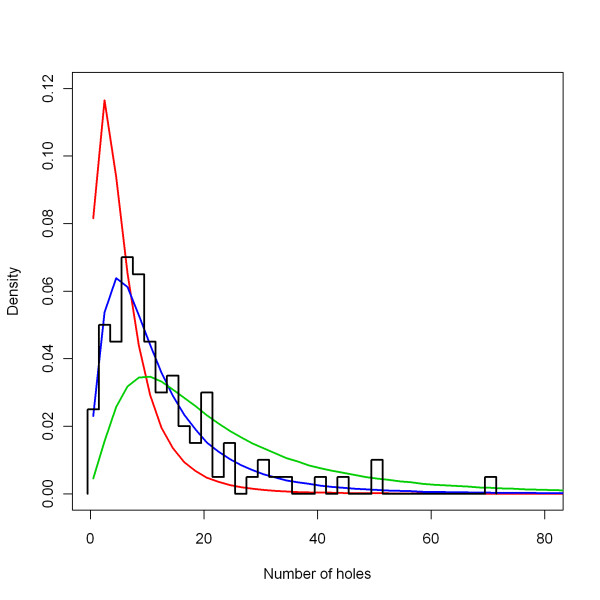
**Distribution of the number of holes after seven years, depending on *holeRate mean***. The black line represents a normalised outline histogram showing the density function of the number of holes as counted by Tami and colleagues [29] in 100 nets. The coloured lines represent the normalised histogram mid-points of the number of holes in simulated nets depending on the *holeRate mean*, which is varied over 0.9 (green), 1.8 (blue) and 3.8 (red), with a constant *holeRate sigma *(see below) of 0.80.

The level of the annual hole formation rate of nets, the *holeRate mean*, was set at 1.8 holes per net per year. This value was based on re-analysis of the data on distribution of the total number of holes in Olyset nets after seven years of use [29], provided by Christian Lengeler. An outline normalised histogram of this distribution is shown in Figure 11. This parameter was varied for the sensitivity analysis. The extreme low parameter value was taken as 0.9 (half of the central value) and the extreme high value was taken as 3.6 (double of the central value). The effects of these parameters are also shown in Figure 11.

#### interventions > ITN > holeRate sigma

The value of the hole formation rate is varied among nets by multiplying with a distribution factor which is log normally distributed with mean one and the standard deviation of the log transformed variable *sigma*). The distribution factor is generated by taking one sample per net from a Gaussian distribution with mean zero and standard deviation one. For each parameter (*holeRate*, *ripRate*, *insecticideDecay *rate), the same sample is multiplied by the respective *sigma *and a constant (*mu*) added such that, once exponentiated, the mean of the variable over nets is one. For *insecticideDecay *rate, this constant can be chosen freely. The transformed sample is then exponentiated to obtain the respective distribution factor. This procedure implies that the distribution of *holeRate*, *ripRate *and insecticide decay rate are supposed to be covariant: nets that are heavily used decay fast both chemically and physically, whereas nets that are gently used decay slowly both chemically and physically. There is some evidence that these are indeed associated [30].

**Figure 12:**
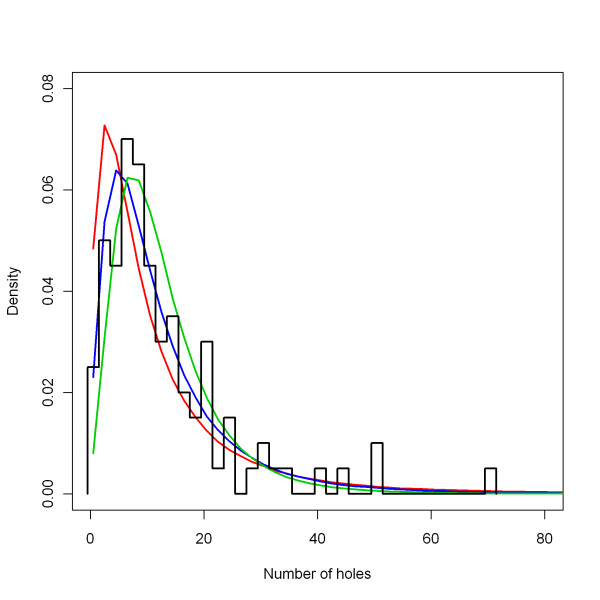
**Distribution of the number of holes after seven years, depending on *holeRate sigma***. The black line represents a normalised outline histogram showing the density function of the number of holes as counted by Tami and colleagues [29] in 100 nets. The coloured lines represent the normalised histogram mid-points of the number of holes in simulated nets depending on the *holeRate sigma*, which is varied over 0.6 (green), 0.8 (blue) and 1.0 (red), with a constant *holeRate mean *(see above) of 1.8.

The central level of the *sigma *parameter of the distribution factor for hole formation rates was set to 0.8. This value was also based on re-analysis of the raw data on distribution of the total number of holes in Olyset nets after seven years of use [29], provided by Christian Lengeler. The *holeRate sigma *parameter was varied for the sensitivity analysis (Figure 12). The extreme low parameter value was taken as 0.60 and the extreme high value was taken as 1.00.

#### interventions > ITN > ripRate mean

The *ripRate mean *value was set equal to the value of the *holeRate mean*. (The ripping process was assumed to be similar to the hole formation process). This parameter was thus varied for the sensitivity analysis, but not independently.

#### interventions > ITN > ripRate sigma

The *riprate sigma *value was set equal to the value of the *holeRate sigma*. (The ripping process was assumed to be similar to the hole formation process). This parameter was thus varied for the sensitivity analysis, but not independently.

#### interventions > ITN > ripFactor value

**Figure 13:**
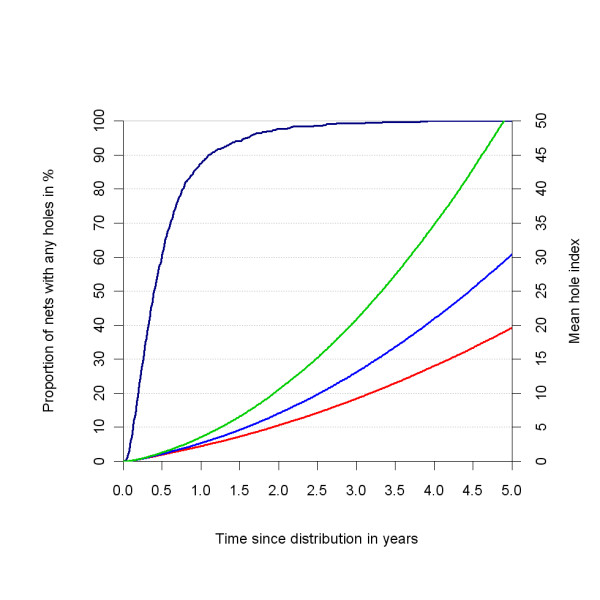
**Mean hole index over time as a function of the *ripFactor value***. Coloured lines plotted on the right hand vertical axis represent the mean hole index depending on the *ripFactor value*, which was varied over 0.6 (green), 0.3 (blue) and 0.15 (red), with all other parameters at central values. The dark blue line plotted on the left hand vertical axis represents the percentage of nets with one or more holes.

The *ripFactor value *expresses how important rips are in increasing the (proportionate) hole index. A net's hole index is the hole count plus the *ripFactor value *multiplied with the cumulative number of rips. With the central values for *holeRate mean*, *ripRate mean*, *holeRate sigma *and *ripRate sigma*, a *ripFactor value *of 0.30 allowed to approximate the upward curve in the mean hole index shown by Kilian and colleagues [31] (See Figure 13). Based on this, the central level of the *ripFactor value *was set to 0.30. Extreme low and extreme high values were chosen of 0.15 and 0.60, respectively.

#### interventions > ITN > initialInsecticide mu

The mean insecticide content of new nets (*initialInsecticide mu*) is set to 68.4 corresponding to the declared deltamethrin content of 68.4 mg.m^-2 ^for long-lasting (incorporated into filaments) insecticidal nets according to WHO interim specification 333/LN/3 [32].

#### interventions > ITN > initialInsecticide sigma

The insecticide concentration of new nets is Gaussian distributed. The standard deviation (*sigma*) was set to 14, based on the interquartile range observed by Kilian and colleagues [31], for Permanet 2nd generation.

#### interventions > ITN > insecticideDecay L and function

**Figure 14:**
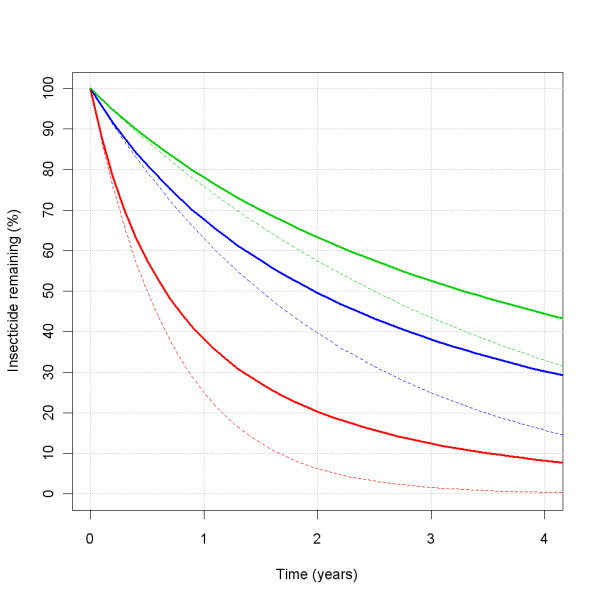
**Mean percentage insecticide remaining over time as a function of *insecticideDecay L***. The coloured lines represent the insecticide remaining over time, as a percentage of the initial concentration, depending on the *insecticideDecay L*, which is varied over 0.5 (red), 1.5 (blue) and 2.5 (green), with a *insecticideDecay sigma *at the central value of 0.8 (solid lines), or at 0 (dotted lines).

The *insecticideDecay function *chosen was "exponential", $\phi_{t}=exp\left(-\frac{t\ln\left(2\right)}{L}\right)$ with *φ_t _*the proportion of the initial insecticide concentration remaining at time *t *(in years). The *insecticideDecay L *parameter then directly translates into the insecticide half-life in years. However, if the decay rate *λ *= ln(2)/*L *is heterogeneous, the mean half-life is longer (Figure 14). The *insecticideDecay L *parameter was varied for the sensitivity analysis. The central level of the *insecticideDecay L *for the decay rate of the insecticide in the nets was taken as 1.5, which, if combined with a central distribution factor *insecticideDecay sigma *of 0.8, yields a mean half-life of about two years. This roughly corresponds to the decay of second generation LLINs [30,31], The extreme low parameter value was taken as 0.5, roughly corresponding to first generation LLINs. The extreme high value was taken as 2.5.

#### interventions > ITN > insecticideDecay sigma (and mu)

**Figure 15:**
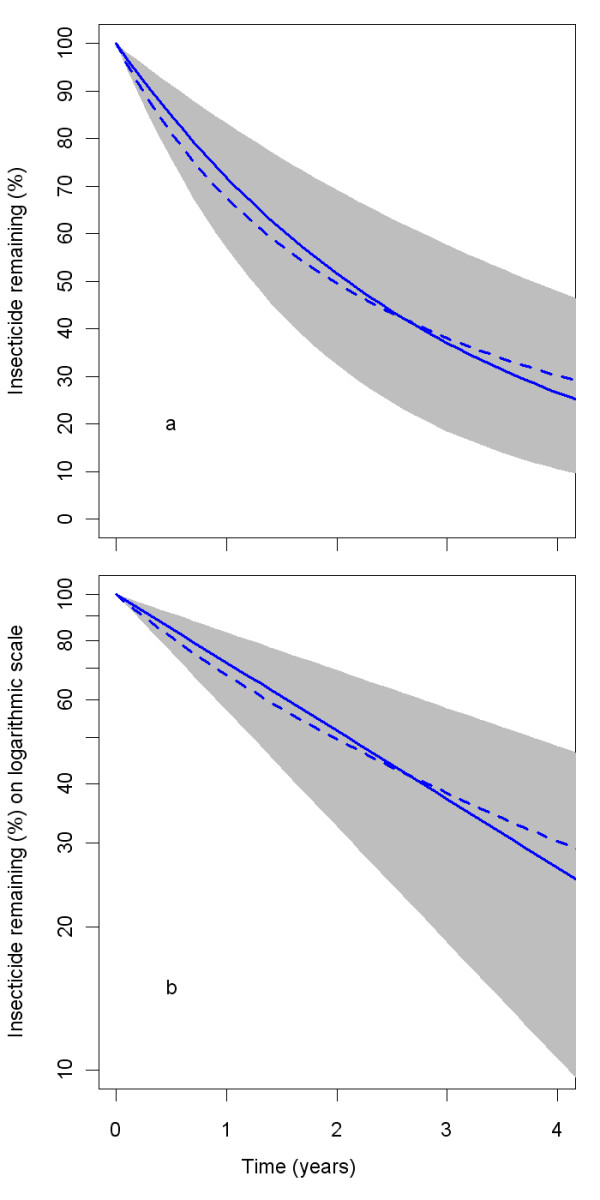
**Percentage insecticide remaining over time with all parameters at central values**. The grey polygon represents the interquartile range, the solid blue line represents the median and the dashed blue line represents the mean.

**Figure 16:**
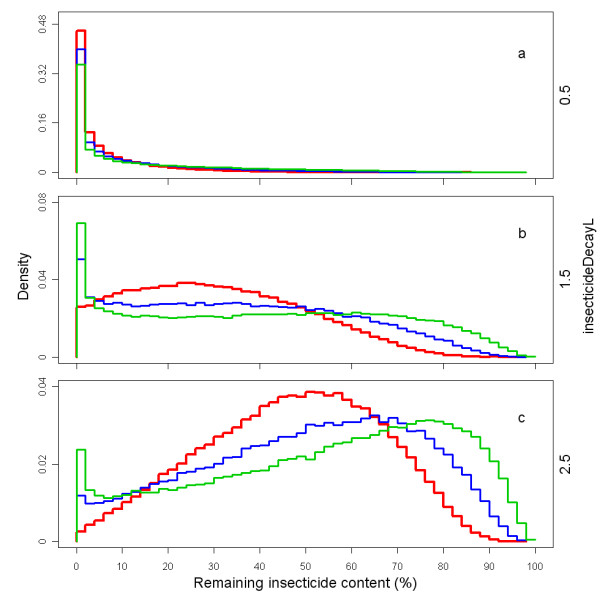
**Distribution of the percentage insecticide remaining of the initial concentration, after 38 months, depending on *insecticideDecay L *and *insecticideDecay sigma***. The coloured lines represent the normalised outline histogram of insecticide remaining as a percentage of the initial concentration in simulated nets depending on the *insecticideDecay sigma*, which is varied over 1.0 (green), 0.8 (blue) and 0.6 (red), and on *insecticideDecay L*, which is varied over 0.5 (a), 1.5 (b) and 2.5 (c).

**Figure 17:**
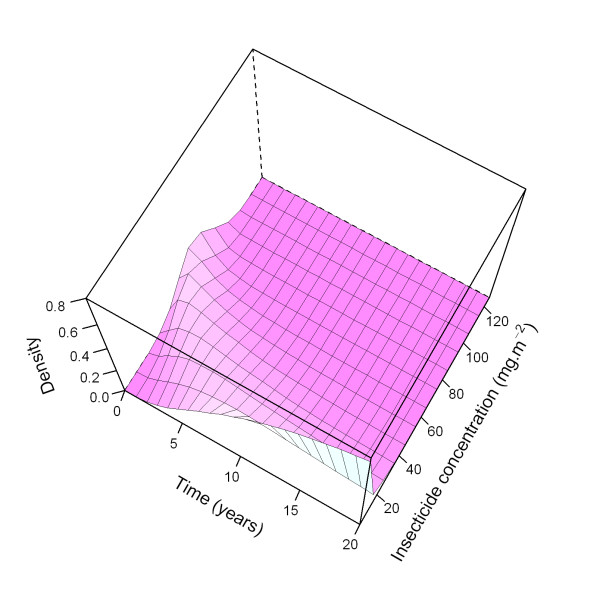
**Density of insecticide concentration over time, with all parameters at central values**. Three dimensional plot with the proportional distribution (density) of nets over categories of insecticide concentration in 10 mg.m^-2 ^intervals, with lines at category mid-points. At time zero, the insecticide concentration is Gaussian distributed over the nets with mean 68.4 and standard deviation 14, but as the insecticide decays over time, the distribution is no longer Gaussian due to heterogeneity in the *insecticideDecay *rate. After 20 years, 77% of the nets has a concentration of 0-10 mg.m^-2^.

The parameters *insecticideDecay mu *and *insecticideDecay sigma *are for the distribution factor (same samples as for the *holeRate *distribution factor). Figure 15 shows how the variation in the insecticide increases over time due to the heterogeneity in the insecticide decay rate *λ*. Such behaviour is also apparent from data presented by Killian and colleagues [31]. The *insecticideDecay sigma *was varied for the sensitivity analysis. The central level of *insecticideDecay sigma *was chosen at 0.8 and *insecticideDecay mu *was chosen such that the mean was equal to one (*insecticideDecay mu *= -0.32 for the central value). The extreme low parameter value was taken as 0.60 and the extreme high value was taken as 1.00. Figure 16 shows the distribution of the percentage insecticide remaining at 38 months. Figure 16a looks similar to Figure 6 presented by Smith and colleagues [33] for first generation LLINs. Figure 17 shows the density of insecticide concentration in nets over time, with all parameters at central values.

#### interventions > ITN > attritionOfNets L and function and k

**Figure 18:**
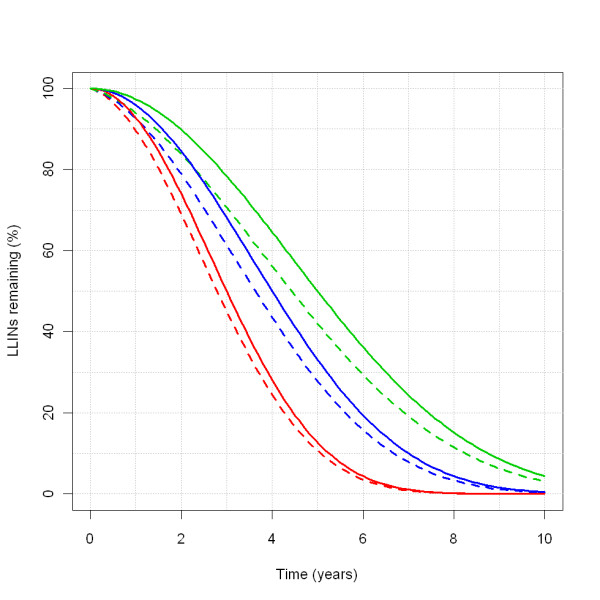
**Percentage of LLINs remaining over time**. The coloured lines represent the LLINs remaining over time after a mass distribution, as a percentage of the initial number, depending on the *attritionOfNets L*, which is varied over 15.579, 20.773 and 25.966, with half-lives of 3 (red), 4 (blue) and 5 (green) years, respectively. Solid lines are for the percentage of nets with as denominator the number of people remaining in the population cohort that initially received LLINs. Dashed lines are for the percentage of nets with as denominator the total (growing) population, presuming an initial coverage of 100%.

The attrition function used is "smooth-compact", $\psi_{t}=exp\left(k-\frac{k}{1-{\left(t/)L\right)}^{2}}\right)$ with *φ_t _*the proportion of the initial net coverage remaining at time *t *(in years). A *k *value of 18 was used. The smooth-compact function with this *k *value was applied by Nakul Chitnis to data on net ownership provided by Albert Kilian (Chitnis and Kilian, personal communications). The *L *parameter was varied for the sensitivity analysis and chosen such that 50% of nets initially distributed had disappeared after 3 (low extreme), 4 (central level) or 5 (high extreme) years. This was at L values of 15.579, 20.773 and 25.966, respectively. It should be noted that from the simulated population, which is kept at a stationary size, people are out-migrated (with their nets) due to population growth. Therefore, the attrition rate of nets per person in the simulated population is slightly higher than the attrition of nets (Figure 18); if the half-life of the attrition of nets would be infinity, with a population growth of 3.47%, the half-life of nets per person in the simulated population would be about 20 years. Population growth may thus explain part of the observed difference in attrition rates between prospective studies (cohort based) and population wide surveys.

#### interventions > ITN > anophelesParams

**Figure 19:**
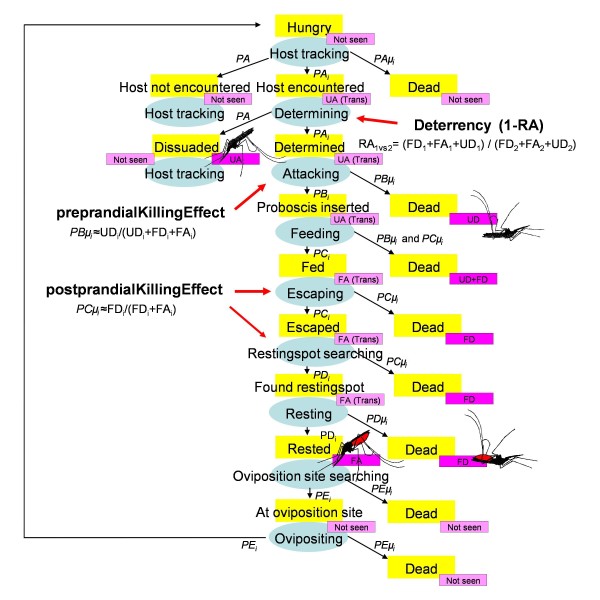
**Flowchart of the mosquito gonotrophic cycle**. Yellow rectangles represent physical states that a mosquito can be in. Blue ovals represent phases that mosquitoes go through to reach a new state. Thin black arrows connect phases (ovals) to resulting states (yellow rectangles). Arrow annotation refers to probabilities as described by Chitnis and colleagues [8]. Purple rectangles represent states of mosquitoes as typically recorded in experimental hut studies: unfed alive (UA); unfed dead (UD); fed alive (FA); fed dead (FD); 'Trans' denotes a transitory state (not an end state as observed in mosquito counts the mornings of experimental hut studies).

For this experiment, effects of ITNs on anopheline mosquitoes were assumed to be equal for all vector species. The mosquito gonotrophic cycle as modelled in the OpenMalaria vector component [8,9] is illustrated in Figure 19. Female mosquitoes enter the hungry state either after emergence or after successfully ovipositing. Then they start host tracking, where they either do not encounter a host (and will continue host tracking the next night) with probability *PA*, die in the process with probability *PA μ*, or encounter a host of type *i *(*PA_i_*).

After encountering a host of type *i*, they determine whether to attack or not (in which case they continue host tracking). These terms are not separately modelled, and included in *PA_i _*and *PA*, respectively. In this model, once a mosquito is determined to attack, it will either successfully feed, or die in the process. Unfed alive (UA) mosquitoes found in experimental huts are thus regarded as those that encountered a host and entered the hut but decided not to attack. This is a simplification of reality, where a mosquito may survive after unsuccessfully trying an attack. Deterrency acts on the determining phase. Deterrency is defined as one minus the relative number of affected mosquitoes (RA_1 vs 2_) of a host of type 1, as compared to another host of type 2. The number of affected mosquitoes is calculated as the sum of fed alive (FA), fed dead (FD) and unfed dead (UD) mosquitoes. A host type that is protected by an ITN will likely have a ratio below one, relative to a similar host type without ITN protection.

A mosquito determined to attack host *i *will either succeed in inserting its proboscis with probability *PB_i_*, or die during the process without inserting its proboscis, with probability *PB μ_i_*. For transmission from mosquito to human, it is important that the proboscis is inserted, and not if blood feeding was successful. However, in OpenMalaria, for simplicity, only blood-fed mosquitoes are assumed to have potentially inoculated hosts with sporozoites. ITNs will have an effect on the pre-prandial killing probability *PB μ_i_*, which can be approximated by the proportion of UD mosquitoes out of the total of determined (unfed dead, and fed dead or alive) mosquitoes.

After proboscis insertion, feeding takes place and the mosquito tries to escape from the host's vicinity, which is successful with probability *PC_i_*, or unsuccessful and the mosquito dies in the process. ITNs will have an effect on the probability of successfully escaping the host after a blood meal, called the post-prandial killing effect. This can be approximated by the proportion of FD mosquitoes out of the total of fed (dead or alive) mosquitoes.

Having escaped the host's vicinity, the mosquito will search for an appropriate resting spot. An ITN might interfere with this (through an excito-repellent effect), but this was not modelled specifically.

After a mosquito finds a resting spot it rests, and survives with probability *PD_i_*. Whereas indoor residual spraying acts during this phase, ITNs were assumed to have no influence here. Experimental hut study procedures will typically collect mosquitoes in the morning (before the resting is complete), and observe alive mosquitoes for an extended period to account for deaths during the resting phase. This is because contact with insecticide picked up during earlier phases may have a delayed effect on mortality.

Rested mosquitoes will search for an oviposition site, oviposit and start another gonotrophic cycle by host tracking.

ITNs give both personal protection by reducing the number of (infectious) bites, and reduce transmission by reducing the mosquito survival per gonotrophic cycle through increased mortality during attack and escape phases, and more time spent host tracking (with associated mortality) due to deterrency.

In the ITN model, new ITNs can decay both physically (formation of holes) and chemically (loss of surface-active ingredient). Published experimental hut studies testing ITNs have typically several (non standard) trial arms, sometimes including no-net control host types (NN), intact untreated net host types (IU), intact insecticide treated net host types (IT), deliberately holed insecticide treated net host types (HT), and holed untreated net host types (HU). These trial arms (Table 1) are of interest as they allow estimating parameter values for the effects of ITNs depending on their (extreme) physical and chemical states. Four references [11-14] were selected, containing six hut experiments with a no-net control and two or more of the other trial arms of interest, and for which the relative proportions of mosquitoes in the UA, UD, FA and FD categories could be retrieved, either from data tables or figures. Note that different authors use different hole numbers and sizes, *e.g*.: 8 holes of 10 × 20 cm [12]; 6 holes of 10 × 5 cm [11]; 6 holes of 10 × 10 cm [14].

**Table 1:** Host types used

Abbreviation	Host type
NN	Person without a net

IT	Person with an intact treated net

IU	Person with an intact untreated net

HT	Person with a holed treated net

HU	Person with a holed untreated net

HsatT	Person with a treated net, holed to saturation

HsatU	Person with an untreated net, holed to saturation

θT	Person with a treated net, with undefined hole index

θU	Person with an untreated net, with undefined hole index

#### interventions > ITN > anophelesParams > deterrency

The relative number of affected mosquitoes in trial arms with a mosquito net as compared to no net, from sources in literature, are displayed in Table 2.

**Table 2:** Relative number of affected mosquitoes by host type

Reference	NN	IT	IU	HT	HU	Holes
[11] EXP 1	1	0.446	0.779			

[11] EXP 2	1			0.682	1.258	6 h, 10 × 5 cm

[11] EXP 3	1			0.549		6 h, 10 × 5 cm

[12]	1	0.552	0.465	0.682	1.025	8 h, 10 × 20 cm

[14]	1	0.769		0.790	1.325	6 h, 10 × 10 cm

[23]	1	0.345	0.410			

geometric mean	1	0.506	0.530	0.670	1.195	

Relative number of affected mosquitoes by host type compared to no net. *h *= holes; *EXP *= experiment; other abbreviations as in Table 1.

Gokool and colleagues [14] and Curtis and colleagues [11] (in their second experiment) found more fed and/or dead *Anopheles gambiae *s.l. in huts with untreated, holed mosquito nets than without nets. Clearly this is an artefact caused by imperfect hut traps, with more mosquitoes escaping from huts without nets, than from huts with nets, where mosquitoes got trapped under the net. It is extremely unlikely that a holed untreated net makes a person more attractive to mosquitoes than when without a net.

The geometric mean data suggest that 0.5 might be a reasonable central value for the RA of IU and IT host types *versus *NN, and 0.67 a good central value for an HT host type. As explained above, if the HU host type is considered not to increase the number of affected mosquitoes, a central value of 1 seems reasonable. These values were used to compute the 'medium' *deterrency *parameter group setting values.

The highest RA values were 0.78, 0.79 and 0.77 for IU, HT, and IT host types, respectively. These values were used to compute the 'low' *deterrency *parameter group setting values.

The lowest RA values were 0.41, 0.55 and 0.35 for IU, HT, and IT host types, respectively. These values were used to compute the 'high' *deterrency *parameter group setting values.

If linear relationships are assumed between the logarithm of the RA and the active insecticide concentration (*p*) and hole index (*h*), where *p *is scaled from zero (no insecticide) to one (maximum active surface concentration for a new ITN) and *h *(which could be expressed as the number of holes, a composite hole index based on number and size of holes, or holed surface in cm^2 ^or as a percentage) is scaled from zero (intact) to one (badly torn net), these could be written as follows:

$$\begin{array}{c} \text{R}{\text{A}}_{i\text{ vs NN}}=\exp\left(\begin{array}{c} \log\left(holeFactor\right)\left(1-h\right)+\\ \log\left(insecticideFactor\right)p+\\ \log\left(interactionFactor\right)\left(1-h\right)p \end{array}\right),\;\text{where:}\;holeFactor=\text{R}{\text{A}}_{\text{IU vs NN}},\\ insecticideFactor=\text{R}{\text{A}}_{\text{HT vs NN}},\;\text{and}\;interactionFactor=\frac{\text{R}{\text{A}}_{\text{IT vs NN}}}{\text{R}{\text{A}}_{\text{IU vs NN}}\times \text{R}{\text{A}}_{\text{HT vs NN}}}. \end{array}$$

However, it was assumed that the relationships between the log of the RA and insecticide and holes are not linear, but increases asymptotically as the number of holes increase, and the insecticide concentration decreases. Thus the following relationship was used:

RAi vs NN=explogholeFactorexp-h×holeScalingFactor+loginsecticideFactor1-exp-p×insecticideScalingFactor+loginteractionFactor exp-h×holeScalingFactor1-exp-p×insecticideScalingFactor

,where *holeScalingFactor *and *insecticideScalingFactor *are scaling factors for the number of holes, and the insecticide concentration, respectively. The *holeScalingFactor *and *insecticideScalingFactor *describe how fast the effect of the hole index, and insecticide concentration, respectively, plateaus. For the 'medium' *deterrency *level this is:

RAivs NN=explog0.5exp-h×holeScalingFactor+log0.671-exp-p×insecticideScalingFactor+log0.50.5×0.67 exp-h×holeScalingFactor1-exp-p×insecticideScalingFactor

**Figure 20:**
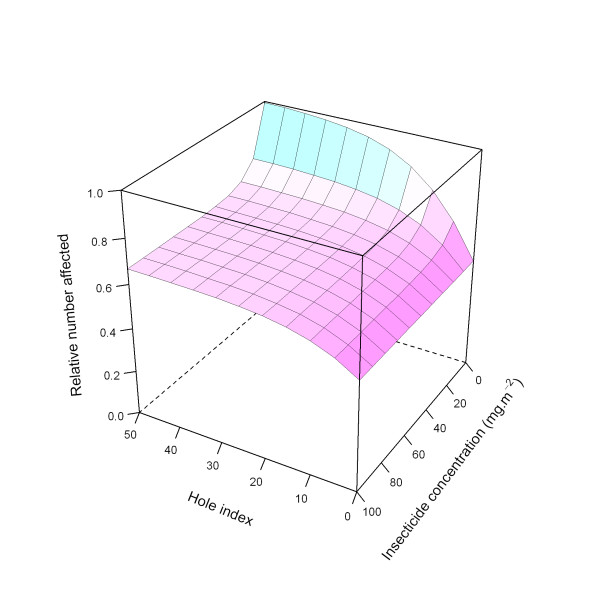
**Modelled relationship between relative number of affected mosquitoes and insecticide concentration and hole index for the 'medium' *deterrency *level**. In this figure, insecticide concentration is varied between 0 and 100 mg.m^-2^, and the hole index is varied between 0 and 50. The scaling factor for holes and insecticide (*holeScalingFactor *and *insecticideScalingFactor*, respectively), are both set to 0.10.

This relationship is illustrated in Figure 20.

**Figure 21:**
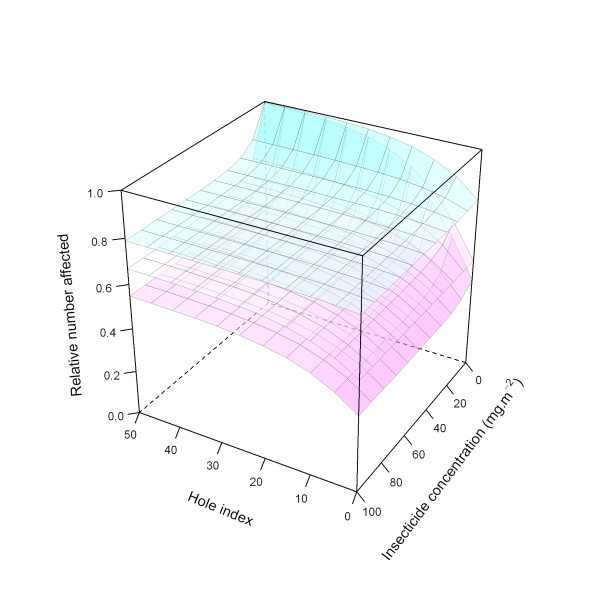
**Modelled relationship between relative number of affected mosquitoes and insecticide concentration and hole index, depending on *deterrency *level**. In this figure, insecticide concentration is varied between 0 and 100 mg.m^-2^, and the hole index is varied between 0 and 50. The scaling factor for holes and insecticide (*holeScalingFactor *and *insecticideScalingFactor*, respectively), are both set to 0.10. Top layer: low *deterrency*; middle layer: medium level *deterrency*, bottom layer: high *deterrency*.

Figure 21 illustrates the ITN effects on *deterrency *for all three modelled levels (low, medium, and high).

Although the *holeScalingFactor *and the *insecticideScalingFactor *can be specified for each ITN effect (*deterrency*, *preprandialKillingEffect *and *postprandialKillingEffect*) separately and for each mosquito (sub) population or species separately, the same *holeScalingFactor *and *insecticideScalingFactor *were used for ITN effects and species in these simulations.

**Figure 22:**
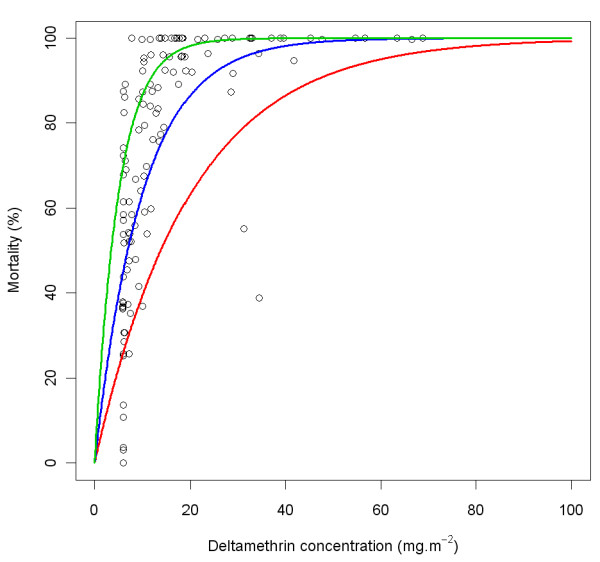
**Relationship of *Anopheles *mortality in WHO cone tests with deltamethrin concentration, and modelled relationship**. Black circles represent observations adapted from Figure 4A, page 40, in the report of the twelfth WHOPES working group meeting [34]. The coloured lines represent modelled relationship mortality = 1 - exp(-*p*×*insecticideScalingFactor*) with *p *the insecticide concentration and with values for *insecticideScalingFactor *of 0.05 (red), 0.1 (blue) and 0.2 (green).

There is little information available in literature to inform the choice for a reasonable value for the *holeScalingFactor *and the *insecticideScalingFactor*. For the *insecticideScalingFactor*, the dose-response curve of insecticide on killing in WHO cone tests [31,34] was used. Figure 22 shows such a relationship for deltamethrin (adapted from [34], Figure 4A, page 40), with the deltamethrin concentration expressed in mg.m^-2 ^(by multiplying the g.kg^-1 ^scale with 85/2.8). Based on the shape of the relationship between the proportion of dead mosquitoes after contact with insecticide in the WHO cone tests, depending on deltamethrin concentration, the value of 0.1 was chosen as the central value, 0.2 was chosen as the high extreme value, and 0.05 was chosen as the low extreme value. This is largely in agreement with experimental hut data presented by Lindsay and colleagues [35] for killing and deterrency. However, the solvent proved to be highly deterrent in that study, making it difficult to use the results to predict the deterrency of aged nets *versus *new nets.

For holes, very little information was available. Carnevale and colleagues [36] published data over a range of physical damage to nets (0.5, 1 and 2% of the net surface) and number of bites, but the relationships varied over the experiments. For this work, the same range of values for the *holeScalingFactor *as used for the *insecticideScalingFactor *was chosen: 0.05, 0.1 and 0.2.

**Figure 23:**
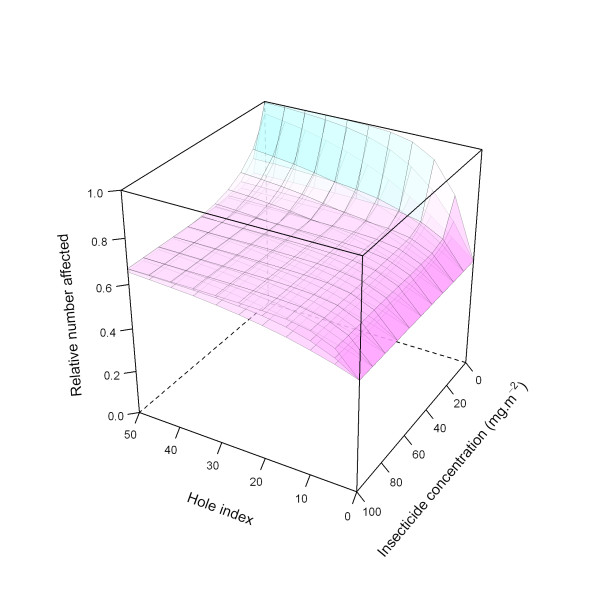
**Modelled relationship between relative number of affected mosquitoes and insecticide concentration and hole index for the 'medium' *deterrency *level, depending on *holeScalingFactor *and *insecticideScalingFactor***. In this figure, insecticide concentration is varied between 0 and 100 mg.m^-2^, and the hole index is varied between 0 and 50. The scaling factor for holes and insecticide (*holeScalingFactor *and *insecticideScalingFactor*, respectively), are both set to 0.20 (top layer), 0.10 (middle layer), and 0.05 (bottom layer).

Figure 23 illustrates the ITN deterrent effects for the medium *deterrency *level, with the three modeled parameter values for the *holeScalingFactor *and the *insecticideScalingFactor *varied simultaneously.

#### interventions > ITN > anophelesParams > preprandialKillingEffect

The pre-prandial killing in trial arms from sources in literature are displayed in Table 3.

**Table 3:** Pre-prandial killing

Reference	NN	IT	IU	HT	HU	Holes
[11] EXP 1	0.170	0.89	0.79			

[11] EXP 2	0.042			0.79	0.145	6 h, 10 × 5 cm

[11] EXP 3	0.156			0.65		6 h, 10 × 5 cm

[12]	0.033	0.62	0.63	0.49	0.120	8 h, 10 × 20 cm

[14]	0.100	0.94		0.71	0.150	6 h, 10 × 10 cm

[23]	0.140	0.81	0.56			

Proportion of unfed dead mosquitoes out of the total of unfed dead, fed dead and fed alive mosquitoes (pre-prandial killing). *h *= holes; *EXP *= experiment; other abbreviations as in Table 1.

In order to calculate a standardised effect, corrected for different mortality in the NN host type arm, probably due to varying environmental and experimental conditions, the following process was adopted:

All proportions were logit transformed and the average value for NN host types was calculated. For each experiment, the difference between the logit NN value and the average logit NN value was subtracted from the logit value of all other treatments. Then, the average for each treatment over all experiments was taken and back transformed into a proportion (Table 4).

**Table 4:** Standardised pre-prandial killing

Reference	NN	IT	IU	HT	HU	Holes
[11] EXP 1	0.09	0.80	0.65			

[11] EXP 2	0.09			0.90	0.28	6 h, 10 × 5 cm

[11] EXP 3	0.09			0.50		6 h, 10 × 5 cm

[12]	0.09	0.83	0.83	0.74	0.29	8 h, 10 × 20 cm

[14]	0.09	0.93		0.69	0.14	6 h, 10 × 10 cm

[23]	0.09	0.72	0.44			

"back transformed mean of logits"	0.09	0.84	0.66	0.73	0.23	

Standardised proportion of unfed dead mosquitoes out of the total of unfed dead, fed dead and fed alive mosquitoes (pre-prandial killing). *h *= holes; *EXP *= experiment; other abbreviations as in Table 1.

The mean proportion killed for NN host types was lowest (0.09), and the mean proportion for HU host types was somewhat higher (0.23). IU host types had a much larger proportion killed (0.66), followed by HT host types (0.73) and IT host types (0.84).

The fact that the average proportion killed for HU host types (0.23) was higher than that of NN host types (0.09) indicates that holed untreated nets do have a small effect on pre-prandial killing, despite the (severe) damage done to the nets. If the nets were damaged even more, the effect would presumably be smaller and eventually, at a saturation point, such a net (host type HsatU), would no longer impede flight of mosquitoes and the pre-prandial mortality would be the same as for NN. It is of interest to estimate the effect of the insecticide at this saturation point, thus for treated nets holed to saturation (HsatT). Presumably, the effect of an HsatT net would be similar to sleeping next to, not under an HT net. If the effect decays with the same shape function (linear or exponential) for both untreated nets and treated nets, this can be calculated.

Let *h *denote the hole index in a net in arbitrary units. The host type *i *can be described by the following letter combinations: NN, HU, IU, IT, HT, HsatT, HsatU, θT or θU (Table 1), depending on the net type the host uses. The letter θ indicates here an undefined hole state. For example, if a linear decay in effect is presumed, if *h*_HsatU _= 1 and *h*_IU _= 0, then *PB μ*_θU _= *PB μ*_IU _- (*PB μ*_IU _- *PB μ_NN_*)*h*_θU _= 0.66 - (0.66 - 0.09)*h*_θU_. Thus, if *PB μ*_HsatU _= 0.23, then $h_{\text{HU}}=\frac{-\left(0.23-0.66\right)}{0.66-0.09}=0.7544$. Then, if *h*_HsaT _= 1, *h*_IT _= 0, and *h*_HT _= *h*_HU _= 0.7544, $PB\mu_{\text{HsatU}}=PB\mu_{\text{IT}}-\frac{PB\mu_{\text{IT}}-PB\mu_{\text{HT}}}{0.7544}=0.694$. Thus the values are 0.66, 0.69, 0.84, and 0.09 for IU, HsatT, IT, and HsatU host types, respectively. These values were used to compute the 'medium' pre-prandial killing parameter group values.

Similarly, the highest values are 0.83, 0.89, 0.93, and 0.09 for IU, HsatT, IT, and HsatU host types, respectively. These values were used to compute the 'high' pre-prandial killing parameter group values.

The lowest values are 0.44, 0.46, 0.72, and 0.09 for IU, HsatT, IT, and HsatU host types, respectively. These values were used to compute the 'low' pre-prandial killing parameter group values.

With *PB μ_i _*the probability of dying before feeding as a result of being committed to biting a host of type *i*, and *p *the active insecticide concentration, with *p *= 1 the maximum active surface concentration for a new ITN, and *p *= 0 for an untreated net, if linear relationships between *PB μ_i _*and *p *and *h *are assumed, these can be written as follows:

$$\begin{array}{c} PB\mu_{i}=baseFactor+holeFactor\left(1-h\right)+insecticideFactor\times p+interactionFactor\left(1-h\right)p,\\ \text{where}\;baseFactor=PB\mu_{\text{HsatU}};\;holeFactor=PB\mu_{\text{IU}}-PB\mu_{\text{HsatU}},\\ insecticideFactor=PB\mu_{\text{HsatT}}-PB\mu_{\text{HsatU}},\\ interactionFactor=PB\mu_{\text{IT}}-PB\mu_{\text{IU}}-PB\mu_{\text{HsatT}}-PB\mu_{\text{HsatU}}. \end{array}$$

For the medium *preprandialKillingEffect *level, this is thus: $PB\mu_{i}=0.09+0.57\left(1-h\right)+0.60p-0.42\left(1-h\right)p$.

Note that for NN, *PB μ_NN _*= 0.09, and that the preprandial killing due to a net is the difference between *PB μ_1 _*(where the subscript 1 indicates a host type with a net) and *PB μ_NN_*.

Similar to the RA, it was assumed that the relationships between the *preprandialKillingEffect *and insecticide and holes are not linear, but decrease asymptotically as the number of holes increase, and increase asymptotically as the insecticide concentration increases. Thus, for the *preprandialKillingEffect*, the following relationship was used:

$$\begin{array}{cc} PB\mu_{i}= & \begin{array}{c} baseFactor+holeFactor\times \exp\left(-h\times holeScalingFactor\right)+\\ insecticideFactor\left(1-\exp\left(-p\times insecticideScalingFactor\right)\right)+\\ interactionFactor\times \exp\left(-h\times holeScalingFactor\right)\left(1-\exp\left(-p\times insecticideScalingFactor\right)\right) \end{array} \end{array}$$

For the medium *preprandialKillingEffect *level this is:

$$\begin{array}{cc} PB\mu_{i}= & \begin{array}{c} 0.09+0.57\times \exp\left(-h\times holeScalingFactor\right)+\\ 0.60\left(1-\exp\left(-p\times insecticideScalingFactor\right)\right)-\\ 0.42\times \exp\left(-h\times holeScalingFactor\right)\left(1-\exp\left(-p\times insecticideScalingFactor\right)\right) \end{array} \end{array}$$

**Figure 24:**
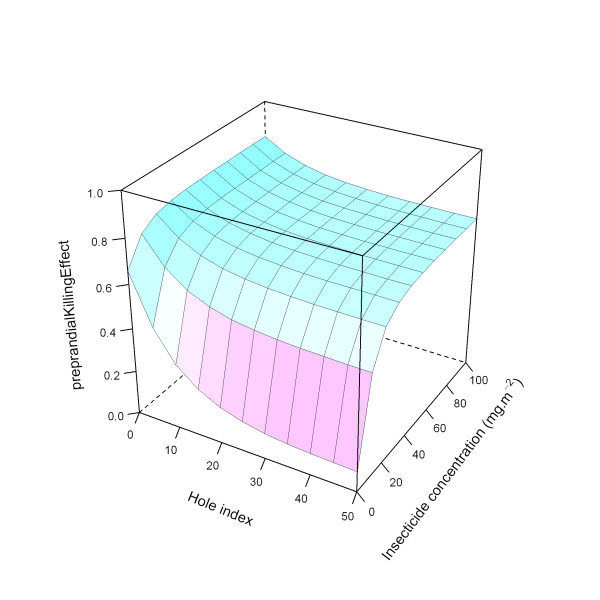
**Modelled relationship between *preprandialKillingEffect *and insecticide concentration and hole index for the 'medium' *preprandial KillingEffect *level**. In this figure, insecticide concentration is varied between 0 and 100 mg.m^-2^, and the hole index is varied between 0 and 50. The scaling factor for holes and insecticide (*holeScalingFactor *and *insecticideScalingFactor*, respectively), are both set to 0.10.

This is illustrated in Figure 24.

**Figure 25:**
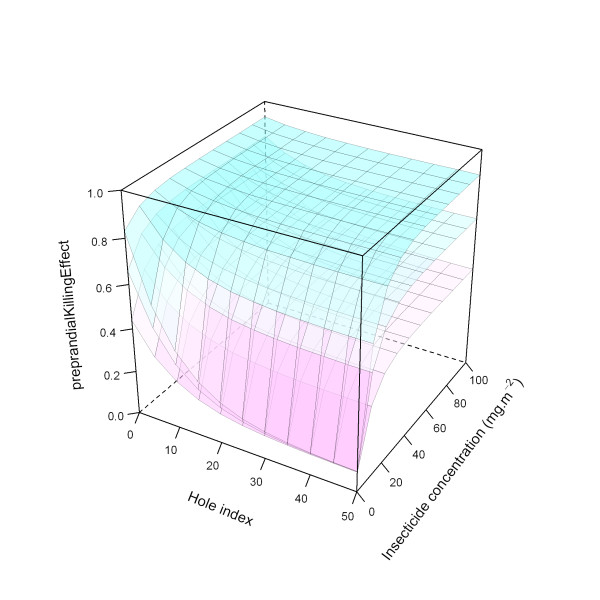
**Modelled relationship between *preprandialKillingEffect *and insecticide concentration and hole index, depending on *preprandialKillingEffect *level**. In this figure, insecticide concentration is varied between 0 and 100 mg.m^-2^, and the hole index is varied between 0 and 50. The scaling factor for holes and insecticide (*holeScalingFactor *and *insecticideScalingFactor*, respectively), are both set to 0.10. Top layer: high *preprandialKillingEffect*; middle layer: medium level *preprandialKillingEffect*, bottom layer: low *preprandialKillingEffect*.

Figure 25 illustrates the ITN effects on pre-prandial killing for all three modelled levels (low, medium, and high).

**Figure 26:**
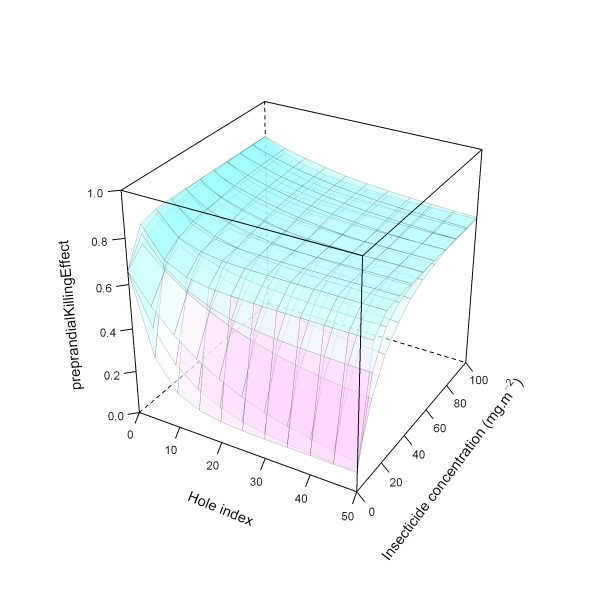
**Modelled relationship between *preprandialKillingEffect *and insecticide concentration and hole index for the 'medium' *preprandialKillingEffect *level, depending on hole scaling factor and insecticide scaling factor**. In this figure, insecticide concentration is varied between 0 and 100 mg.m^-2^, and the hole index is varied between 0 and 50. The scaling factor for holes and insecticide (*holeScalingFactor *and *insecticideScalingFactor*, respectively), are both set to 0.20 (top layer), 0.10 (middle layer), and 0.05 (bottom layer).

Figure 26 illustrates the ITN effects on pre-prandial killing for the medium level, with the three modelled parameter values for the *holeScalingFactor *and the *insecticideScalingFactor*, here both varied simultaneously.

#### interventions > ITN > anophelesParams > postprandialKillingEffect

The post-prandial killing in trial arms from sources in literature are displayed in Table 5, and the standardised values (see pre-prandial killing) are displayed in Table 6.

**Table 5:** Post-prandial killing

Reference	NN	IT	IU	HT	HU	Holes
[11] EXP 1	0.13	0.70	0.064			

[11] EXP 2	0.25			0.73	0.135	6 h, 10 × 5 cm

[11] EXP 3	0.44			0.89		6 h, 10 × 5 cm

[12]	0.07	0.53	0.016	0.56	0.04	8 h, 10 × 20 cm

[14]	0.18	0.60		0.80	0.14	6 h, 10 × 10 cm

[23]	0.013	0.18	0.05			

Proportion of fed dead mosquitoes out of the total of fed dead and fed alive mosquitoes (post-prandial killing). *h *= holes; *EXP *= experiment; other abbreviations as in Table 1.

**Table 6:** Standardised post-prandial killing

Reference	NN	IT	IU	HT	HU	Holes
[11] EXP 1	0.13	0.69	0.06			

[11] EXP 2	0.13			0.54	0.06	6 h, 10 × 5 cm

[11] EXP 3	0.13			0.60		6 h, 10 × 5 cm

[12]	0.13	0.68	0.03	0.71	0.07	8 h, 10 × 20 cm

[14]	0.13	0.49		0.72	0.10	6 h, 10 × 10 cm

[23]	0.13	0.70	0.36			

"back transformed mean of logits"	0.13	0.65	0.09	0.64	0.08	

Standardised proportion of fed dead mosquitoes out of the total of fed dead and fed alive mosquitoes (post-prandial killing). *h *= holes; *EXP *= experiment; other abbreviations as in Table 1.

The mean post-prandial killing for the HU host type (0.08) was similar to that of the IU host type (0.09), Similarly, the mean post-prandial killing for the HT host type (0.64) was similar to that of the IT host type (0.65). For the mean post-prandial killing effect, the physical net state (intact or badly holed) thus has a minimal effect on post-prandial killing.

The HU and IU host type values were slightly lower than the value for NN, which is counter-intuitive, because nets are not expected to protect against mosquito mortality.

The values 0.10, 0.65, 0.0.65, and 0.10 are proposed for IU, HsatT, IT, and HsatU host types, respectively. These values were used to compute the 'medium' *postprandialKillingEffect *parameter group setting values.

Similarly, the values 0.36, 0.70, 0.70, and 0.10 for IU, HsatT, IT, and HsatU host types, respectively, were used to compute the 'high' *postprandialKillingEffect *parameter group setting values.

The values 0.10, 0.50, 0.50, and 0.10 for IU, HsatT, IT, and HsatU host types, respectively, were used to compute the 'low' *postprandialKillingEffect *parameter group setting values.

**Figure 27:**
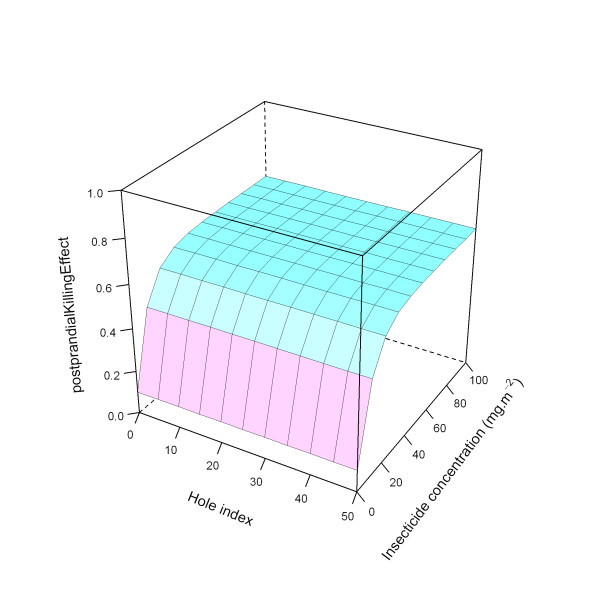
**Modelled relationship between pre-prandial killing and insecticide concentration and hole index for the 'medium' *postprandialKillingEffect *level**. In this figure, insecticide concentration is varied between 0 and 100 mg.m^-2^, and the hole index is varied between 0 and 50. The scaling factor for holes and insecticide (*holeScalingFactor *and *insecticideScalingFactor*, respectively), are both set to 0.10.

Similarly to *preprandialKillingEffect*, the following relationship was used:

$$\begin{array}{cc} PC\mu_{i}= & \begin{array}{c} baseFactor+holeFactor\times \exp\left(-h\times holeScalingFactor\right)+\\ insecticideFactor\left(1-\exp\left(-p\times insecticideScalingFactor\right)\right)+\\ interactionFactor\times \exp\left(-h\times holeScalingFactor\right)\left(1-\exp\left(-p\times insecticideScalingFactor\right)\right) \end{array} \end{array}$$

with *PC μ_i _*the probability of dying (for a mosquito) after biting as a result of the biting process on host type *i*. For example for the medium *postprandialKillingEffect *level this is:

*PC μ_i _*= 0.10 + 0 + 0.55(1-exp(-*p × insecticideScalingFactor*))+0. This is illustrated in Figure 27.

**Figure 28:**
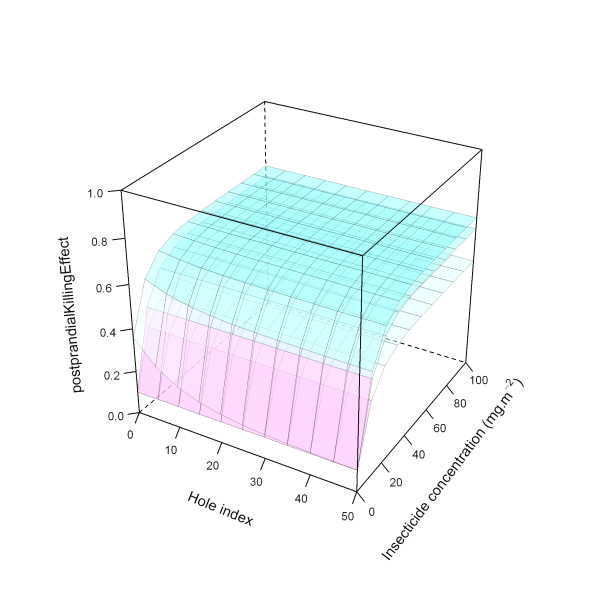
**Modelled relationship between *postprandialKillingEffect *and insecticide concentration and hole index, depending on *postprandialKillingEffect *level**. In this figure, insecticide concentration is varied between 0 and 100 mg.m^-2^, and the hole index is varied between 0 and 50. The scaling factor for holes and insecticide (*holeScalingFactor *and *insecticideScalingFactor*, respectively), are both set to 0.10. Top layer: high *postprandialKillingEffect*; middle layer: medium level *postprandialKillingEffect*, bottom layer: low *postprandialKillingEffect*.

Figure 28 illustrates the ITN effects on post-prandial killing for all three modelled levels (low, medium, and high).

**Figure 29:**
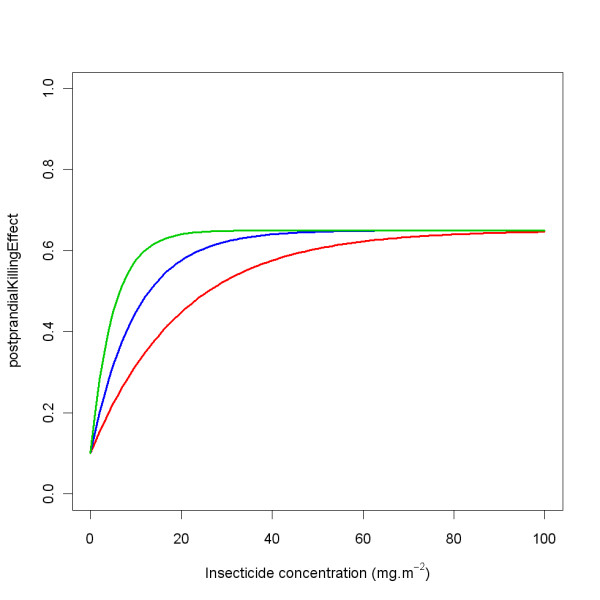
**Modelled relationship between the *postprandialKillingEffect *and insecticide concentration for the 'medium' *postprandialKillingEffect *level, depending on insecticide scaling factor**. In this figure, insecticide concentration is varied between 0 and 100 mg.m^-2^. The scaling factor for insecticide (*insecticideScalingFactor*), is set to 0.20 (green line), 0.10 (blue line), and 0.05 (red line).

Figure 29 illustrates the ITN effects on post-prandial killing for the medium level, with the three modelled parameter values for the *holeScalingFactor *and the *insecticideScalingFactor*, here both varied simultaneously.

#### interventions > ITN > timed coverage

**Figure 30:**
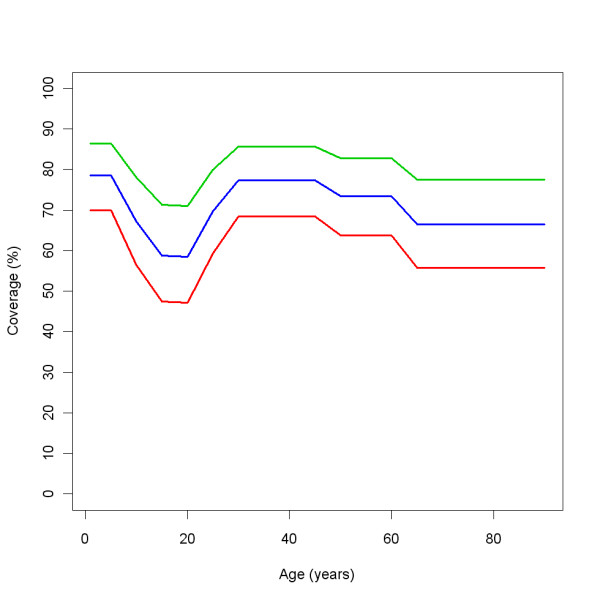
**Coverage depending on age**. The coloured lines represent the LLIN coverage (use) directly after a mass distribution, depending on age, at 60 (red), 70 (blue) and 80 (green) average coverage, with the population structure used in the simulations (see Additional file 1).

The central level of the *coverage*, which describes the proportion of people that receive a net during mass distribution of nets, was set at 0.7 (70%). This parameter was varied for the sensitivity analysis. The low extreme level was set at 0.6, and the high extreme level at 0.8. Eighty percent is a reasonable figure for mass distributions targeting the entire population (de Savigny, personal communication). The nets were not randomly distributed over the population, but spread over the population according to age-use curves (Figure 30). Data from seven countries [37] was logit-transformed, and the average over all countries was taken for each age group. A constant was added such that, when the sum of the age group specific value and constant was back transformed and multiplied with the size of the age group, the total *coverage *was at the required level over the whole population (all age groups). The mass delivery was done in the last five-day step of the fifth year into the simulation run. The 0 *coverage *level was used as comparator.

#### interventions > importedInfections

From time step zero onwards, 10 infections per 1,000 population per year were imported by stochastically infecting individuals in the population. This was done to ensure that malaria would not be eliminated from the simulated population, which might overestimate the protective effect of an intervention.

Thus, even if an intervention provides full protection to the entire population, 1% of the population will be infected once per year. These infections do not necessarily develop into disease episodes. These could be seen as infections obtained while travelling to a malarious area.

#### healthSystem

The "Tanzania ACT" health system was used, described elsewhere [38].

#### entomology > annualEIR

In the OpenMalaria schema version used (Schema 29), during the warm-up phase of the simulation run, the mosquito emergence rate is scaled such that the average annual malaria transmission to an adult (expressed as the number infectious bites per adult per annum) is approximately equal to this parameter value. Post warm-up, in the absence of interventions, this annual EIR is approximately constant. However, interventions such as ITN distributions will affect the transmission. Hence, the *annualEIR *is referred to is the pre-intervention EIR. For the sensitivity analysis, the *annualEIR *was varied between 8, 16, and 32 ibpapa. For the in-depth analysis, also values of 2, 4, 64, 128 and 256 ibpapa were used, covering a low to high range of stable malaria transmission.

#### entomology > mode and name

The "Namawala" seasonality and relative species abundance was used [39], with a dynamic transmission mode. Briefly, there are three species (*Anopheles gambiae *s.s., *Anopheles arabiensis *and *Anopheles funestus*. *An. gambiae *s.s. and *An. arabiensis *had the same seasonality. The seasonality of the natural logarithm of the observed density was fitted with a third order Fourier series, thus with a mean term, two terms for the annual frequency and two terms for the semi-annual frequency. The other parameter values in this section are described elsewhere [8].

#### model

Here, the specification for the "base" model [7,25,40-42] is given. All scenarios included in the experiment were run with 14 different malaria model variants [10].

## Supplementary Material

Additional file 1 **Central scenario**. Annotated OpenMalaria scenario file. Click here for file
